# Transcriptomic landscape of lncRNAs in inflammatory bowel disease

**DOI:** 10.1186/s13073-015-0162-2

**Published:** 2015-05-13

**Authors:** Aashiq H Mirza, Claus HB Berthelsen, Stefan E Seemann, Xiaoyong Pan, Klaus S Frederiksen, Mogens Vilien, Jan Gorodkin, Flemming Pociot

**Affiliations:** Center for non-coding RNA in Technology and Health, University of Copenhagen, Frederiksberg, 1870 Denmark; Department of Pediatrics E, Copenhagen Diabetes Research Center (CPH-DIRECT), Herlev University Hospital, Herlev, 2730 Denmark; Faculty of Health and Medical Sciences, University of Copenhagen, Copenhagen, 2200 Denmark; Department of Surgery, North Zealand Hospital, Hillerød, 3400 Denmark; The Novo Nordisk Foundation Center for Protein Research, University of Copenhagen, Copenhagen, 2200 Denmark; Department of Obesity Biology, Novo Nordisk, Måløv, 2760 Denmark; Department of Molecular Genetics, Novo Nordisk, Måløv, 2760 Denmark

## Abstract

**Background:**

Inflammatory bowel disease (IBD) is a complex multi-factorial inflammatory disease with Crohn’s disease (CD) and ulcerative colitis (UC) being the two most common forms. A number of transcriptional profiling studies have provided compelling evidence that describe the role of protein-coding genes and microRNAs in modulating the immune responses in IBD.

**Methods:**

In the present study, we performed a genome-wide transcriptome profiling of lncRNAs and protein-coding genes in 96 colon pinch biopsies (inflamed and non-inflamed) extracted from multiple colonic locations from 45 patients (CD = 13, UC = 20, controls = 12) using an expression microarray platform.

**Results:**

In our study, we identified widespread dysregulation of lncRNAs and protein-coding genes in both inflamed and non-inflamed CD and UC compared to the healthy controls. In cases of inflamed CD and UC, we identified 438 and 745 differentially expressed lncRNAs, respectively, while in cases of the non-inflamed CD and UC, we identified 12 and 19 differentially expressed lncRNAs, respectively. We also observed significant enrichment (*P*-value <0.001, Pearson’s Chi-squared test) for 96 differentially expressed lncRNAs and 154 protein-coding genes within the IBD susceptibility loci. Furthermore, we found strong positive expression correlations for the intersecting and *cis*-neighboring differentially expressed IBD loci-associated lncRNA-protein-coding gene pairs. The functional annotation analysis of differentially expressed genes revealed their involvement in the immune response, pro-inflammatory cytokine activity and MHC protein complex.

**Conclusions:**

The lncRNA expression profiling in both inflamed and non-inflamed CD and UC successfully stratified IBD patients from the healthy controls. Taken together, the identified lncRNA transcriptional signature along with clinically relevant parameters suggest their potential as biomarkers in IBD.

**Electronic supplementary material:**

The online version of this article (doi:10.1186/s13073-015-0162-2) contains supplementary material, which is available to authorized users.

## Background

Inflammatory bowel diseases (IBDs) are idiopathic chronic relapsing inflammatory conditions of the gastrointestinal tract. Crohn’s disease (CD) and ulcerative colitis (UC) are two most common forms of the IBD. IBD is emerging as a global disease with its incidence and prevalence differentially increasing geographically around the world. Accumulating evidence suggests that IBDs result from the complex interplay between genetic, immunologic, and modifiable environmental factors [1] in a genetically susceptible host against a subset of gut commensal microbiota [2-4].

CD is characterized by intestinal inflammation in a discontinuous fashion and involves any part of the gastrointestinal tract, although in most cases the terminal ileum and/or colon is affected. A transmural pattern of inflammation is a hallmark of CD accompanied by other pathophysiological complications like thickened submucosa, intestinal fibrosis, fissuring ulceration in highly active disease, non-caseating granulomas, strictures, abscesses and fistulas [3]. By contrast, UC involves only the rectum and colon, and is characterized by superficial inflammation that is restricted to the mucosa and submucosa with the presence of cryptitis and crypt abscesses. Disease activity in both CD and UC is typically relapsing and remitting and both conditions are often difficult to diagnose because of idiosyncrasies in the presentation of overlapping and distinct clinical and pathological features [2,3]. Characteristically, diagnosis of either CD or UC is based on a number of findings, including clinical symptoms, endoscopic features, radiologic tests, and biopsy histology.

According to recent meta-analysis of IBD genome-wide association studies data, the number of confirmed genetic loci associated with risk for IBD has increased to 163, with 110 shared between CD and UC, 30 CD-specific and 23 UC-specific. Interestingly, an overwhelming majority of these IBD loci are located in the noncoding intergenic and intronic regions [5]. Most overlap regulatory elements and consequently are likely to influence gene regulation. Findings from our recent studies have demonstrated that a large number of annotated long non-coding RNAs (lncRNAs), including novel evolutionarily conserved structured RNA motifs with regulatory potential [6] (SE Seemann *et al*., unpublished observations), overlap the IBD loci. Consistent with our findings, another recent study elegantly revealed that IBD loci overlap active regulatory regions in primary intestinal epithelium and immune cells and also were found significantly enriched within these active regulatory regions [7].

Several transcriptome profiling studies have provided compelling evidence describing the role of protein-coding and non-coding RNAs (ncRNAs), such as microRNAs, in modulating immune responses in IBD [8-15]. In murine models, loss of endogenous intestinal microRNAs is known to cause impairment of epithelial barrier function that results in acute inflammation [16]. Several studies have explored clinical differences between CD and UC based on transcriptional regulation [17,18]. Recently, Granlund *et al*. [9] demonstrated lack of major differences between CD and UC based on protein-coding gene expression profiling in IBD. In contrast, expression profiling of colon biopsies from IBD patients allude to differential diagnosis of CD and UC based on transcriptional signatures associated with intestinal inflammation [19].

LncRNAs have emerged as important regulators of gene expression, with an accumulating body of evidence linking lncRNAs to a plethora of human pathologies, including inflammatory diseases [20]. Although, the precise role of lncRNAs in intestinal diseases remains poorly understood, evidence from recent studies indicates that lncRNAs might be playing a crucial role in inflammatory cascades. Indeed, a preponderance of emerging evidence from a number of studies demonstrates important roles for lncRNAs in regulating gene expression within the immune system. Nevertheless, identification of IBD susceptibility loci has afforded limited success in translating results from gene expression studies to advance our knowledge and understanding of IBD pathophysiology. In particular, details about the initiation, propagation and maintenance of the lingering inflammation in IBD remains unclear. Furthermore, earlier transcriptomic studies in IBD have mostly focused on the protein-coding genes, with few profiling studies focusing on microRNAs. However, no study has explored the genome-wide expression profile of lncRNAs in IBD.

In the present study, transcriptomic profiling of lncRNAs and protein-coding genes from colon pinch biopsies of IBD patients was performed using an expression microarray platform. Our results identified widespread dysregulation of lncRNAs and protein-coding gene expression in both CD and UC. Notably, the differential transcriptomic signatures of lncRNAs and protein-coding genes in inflamed CD (iCD) and inflamed UC (iUC) enabled clear stratification of the CD and UC phenotypes. These data indicate that lncRNAs could potentially be used as predictive biomarkers in IBD.

## Methods

### Sample collection for patients and controls

All the patient samples were collected from an IBD cohort at North Zealand Hospital, Hillerød, Denmark. Subjects were required to meet the Copenhagen criteria for CD or UC. Participants recruited for the study were patients admitted to the Department of Gastroenterology for colonoscopy who were diagnosed either with CD or UC, or were admitted to the clinic for diagnostic colonoscopy because of symptoms unrelated to the IBD. Written informed consent from all the participants in the study was acquired prior to the collection of samples and medical history. In total, 90 biopsies were collected from 45 individuals (13 CD, 20 UC patients and 12 healthy controls). Subjects were included as normal controls only after all clinical examinations had concluded no signs of autoimmune or inflammatory disease. For the IBD groups (CD and UC), one to five endoscopic pinch biopsies were extracted from the macroscopically most inflamed mucosa (iCD/iUC) and adjacent non-inflamed (niCD/niUC) mucosa within colon (transverse, descending, sigmoid), ileum, transverse ileum and rectum for the CD patients, and colon sigmoid and rectum for the UC patients. For the control group, one to five biopsies were taken from the same locations as in the CD group, except for two samples (G3_G1 and 60_G1) that were extracted from the duodenal bulb. All biopsies were placed in RNA*later* solution (QIAGEN, Hilden, Germany), and stored for later downstream use. The study was approved by the Regional Ethical Committee (H-4-2012-030). The inflammation status of biopsies was confirmed by histologic examination and features of chronic intestinal inflammation for each patient were scored using a previously described scoring system for UC [21] and CD [22]. The pathologists were blinded to the status of inflammation. Additionally, we also tested expression of a panel of 26 pro-inflammatory markers (cytokines, interleukins, metalloproteases) using quantitative real-time PCR (qPCR; Fluidigm platform) to confirm the inflammation status of biopsies (data not shown) prior to the microarray analysis.

### RNA extraction and quality control

Total RNA was extracted from biopsies stored in RNA*later* using RNeasy Mini Kit (QIAGEN) according to the manufacturer’s instructions. Briefly, the biopsy samples were homogenized in lysis buffer with 1.4 mm ceramic beads (MO BIO Laboratories, Carlsbad, CA, USA) using a Thermo Savant FastPrep FP120 Homogenizer (Carlsbad, CA, USA) for 30 s at a speed of 4 m/s. All the remaining steps of the protocol were performed according to the manufacturer’s recommendations. To remove traces of genomic DNA, samples were treated with DNase I (QIAGEN). RNA was finally eluted with nuclease-free water supplied with the kit. The quantity and purity of isolated RNA was determined by UV absorbance using a NanoDrop 2000 Spectrophotometer (Thermo Scientific, Wilmington, DE, USA), and the integrity of RNA was assessed by analysis of rRNA band integrity on an Agilent 2100 Bioanalyzer RNA 6000 LabChip kit (Agilent Technologies). Only RNA samples with RNA integrity number (RIN) >7 were used for the microarray experiments.

### Microarray hybridization

Total RNA (100 ng) was labeled using a LowInputQuick Amp Labeling kit v6.5 (Agilent 5190-2305) following the manufacturer’s instructions. Briefly, mRNA was reverse transcribed in the presence of T7-oligo-dT primer to synthesize cDNA. The cDNA was then *in vitro* transcribed with T7 RNA polymerase in the presence of Cy3-CTP to generate labeled cRNA. The labeled cRNA was hybridized to the Agilent Custom 8x60K format lncRNA expression microarray (AMADID 047718, based on Gencode v.15 catalog of human long ncRNAs, probe length of 60 nucleotides) according to the manufacturer’s protocol. Finally, the arrays were washed, and scanned on an Agilent G2565CA microarray scanner at 100% PMT (photomultiplier tube) and 3 μm resolution. Intensity data were extracted using the Feature Extraction software (Agilent). More detailed and general information about the array can also be found on the GENCODE website [23]. The raw microarray data reported in this manuscript have been deposited in the Gene Expression Omnibus (GEO) database with accession number GSE67106.

### Statistical analyses

Raw data were corrected for background noise using the normexp method [24]. To assure comparability across samples, we used quantile normalization [25] (unpublished observations). Median intensity was taken between technical replicates after checking pairwise Pearson correlation coefficients (r^2^ ≥ 0.98). Differential expression analysis was carried out on non-control probes with an empirical Bayes approach on linear models (LIMMA) [26]. Principal component analysis (PCA) was employed for the initial interpretation of the data. In total, we made seven comparisons to identify differentially expressed genes (iCD versus control, iUC versus control, iCD versus niCD, iUC versus niUC, niCD versus control, niUC versus control and iCD versus iUC) (Tables S1, S2, S3, and S4 in Additional file 1). *P*-values were adjusted for multiple comparisons using the false discovery rate (FDR) correction [27]. Differentially expressed genes were identified using the double-filtering criterion: adjusted *P*-value (FDR) <0.05 and an absolute fold change (absolute FC) >1.5. For transcripts targeted by two probes, only those probes that were changing in the same direction and the probes with highest FC values were retained for further analysis. All statistical analyses were performed with Bioconductor in the R statistical environment [28].

### Validation of differentially expressed genes by quantitative real-time PCR

The expression of differentially expressed genes from microarray experiments was validated by qPCR using hydrolysis probe-based inventoried and custom designed PrimeTime qPCR 5′ Nuclease assays procured from Integrated DNA Technologies, Coralville, IA, USA. The double-quenched hydrolysis probes with 5′ FAM fluorophore, a 3′ IBFQ quencher, and an internal ZEN™ quencher were used for all assays. From the list of the top differentially expressed genes from the different comparisons, six up- and six down-regulated genes were selected for their expression validation by qPCR in a subset of samples used for the microarray experiments. Three up-regulated (*DUOXA2*, *CHI3L1*, *DST*), and three down-regulated (*PCK1*, *KCNK10*, and *SERPINB3*) protein-coding genes were validated, and three up-regulated (*MMP12*, *RP11-731 F5.2*, *AC007182.6*) and three down-regulated (*DPP10-AS1*, *CDKN2B-AS1*, and *AL928742.12*) lncRNA genes were validated. In addition, expression of the protein-coding gene *DUOX2* was also measured by qPCR. All cDNAs were prepared using 750 ng of DNA-free RNA using an iScript™ cDNA synthesis kit (BioRad, Hercules, CA, USA) with a mixture of random and oligo(dT) primers following the manufacturer’s instructions. Real-time PCR was performed with 7.5 ng of cDNA per well template for all the protein-coding genes and lncRNAs with Brilliant III Ultra-Fast QPCR Master Mix (Agilent Technologies). For PCR amplification, the following thermal profile was used: 3 minutes at 95°C; 40 × (5 s at 95°C, 10 s at 60°C). Expression of each lncRNA and protein-coding gene tested was represented as a FC using the 2^-∆∆CT^ method. *GAPDH* was used as the reference gene.

### Identification of inflammatory bowel disease loci associated lncRNAs

All IBD loci marker SNPs and associated genes were retrieved from ImmunoBase [29]. In total, 233 unique marker SNPs for IBD, CD, and UC regions were retrieved and mapped to the 22,007 lncRNAs (Gencode v.15) using the intersect feature of BedTools [30]. The susceptibility locus for IBD was defined based on a 500 kb long genomic region with the IBD marker SNP in the middle. The differentially expressed lncRNAs from five comparisons (iCD versus control, iUC versus control, iCD versus niCD, iUC versus niUC, and iCD versus iUC) were mapped to the IBD loci to identify the IBD loci-associated lncRNAs. Regulatory evidence for the IBD-associated SNPs was retrieved from Mokry *et al*. [7] and RegulomeDB [31].

### Functional annotation and Gene Ontology analysis of differentially expressed lncRNAs

For the differentially expressed lncRNAs, the nearest protein-coding neighbors within a span of <10 kb were identified. For the antisense overlapping or intronic overlapping lncRNAs, intersecting protein-coding gene(s) were identified using the intersect feature of BedTools [30]. The PANTHER (protein annotation through evolutionary relationship) classification system [32] was used to perform functional annotation and Gene Ontology (GO) analysis of genes that overlap with or are neighbors of the differentially expressed lncRNAs. Likewise, for the IBD loci-associated lncRNAs, GO analysis was performed using the above described nearest neighbor approach. The enrichment for over-represented GO functional terms was calculated based on the binomial test in PANTHER.

### Sample classification using Support Vector Machines based on differentially expressed genes identified by LIMMA

Support Vector Machines (SVM) [33] was used for classifying the CD and UC cases from the controls based on differentially expressed genes identified by LIMMA in five comparisons (iCD versus control, iCD versus niCD, iUC versus control, iUC versus niUC and iCD versus iUC). SVM classification was applied to all five comparisons using leave-one-out cross-validation for differentially expressed lncRNAs and protein-coding genes. To explore the effect of various clinical parameters (age, sex, smoking, disease index and biopsy location) on overall disease outcome, we used the following linear regression function:

$$ y = err + w1* age + w2* sex + w3* smoking + w4* disease\  index + w{5}^{*} biopsy\  location $$

Here, y = 1 for the iCD or iUC disease phenotype and 0 for the rest of the samples. For the clinical parameters age and disease index we used original values, while for sex and smoking we used the following binary outcomes: male = 1, female = 0 and smoker =1, non-smoker = 0. For the six biopsy locations, we used values ranging from 0 to 5. To control any input bias, the same analysis was performed on a randomized lncRNA gene list with the same number of genes as the total differentially expressed lncRNA genes. The feature values were normalized to values ranging from 0 to 1 using (x - Minimum)/(Maximum - Minimum). Linear regression was applied using the Scikit-learn [34] package in Python and the least squares method was used for optimization in our analysis. Furthermore, differentially expressed genes identified by LIMMA were verified by the SVM-recursive feature elimination (SVM-RFE) method [35]. SVM-RFE recursively prunes genes whose absolute weights are the smallest until the desired number of features is reached. For each comparison, we used SVM-RFE to identify the same number of differentially expressed genes as identified by LIMMA.

### Co-expression network analysis

To identify CD- and UC-specific network clusters (modules) based on highly correlated genes, the weighted correlation network analysis (WGCNA) method was used [36]. We used the normalized expression data as input and removed the outlier samples. The clinical parameters were represented as follows: numeric for age and disease, binary for sex, ethnicity (three categories), smoking (four categories), clinical subgroup (five categories), and biopsy location (six categories). The standard procedure of WGCNA was applied for network construction and module identification. The trait-based gene significance measure is defined as the absolute correlation and correlation test *P*-value between the trait and the gene expression profile. GO analysis of modules was performed with the GOstats package in R [37] using adjusted *P*-value <0.001. We controlled for study bias in the GO analysis by running the same analysis for randomized gene sets with the same module sizes.

## Results

**Figure 1:**
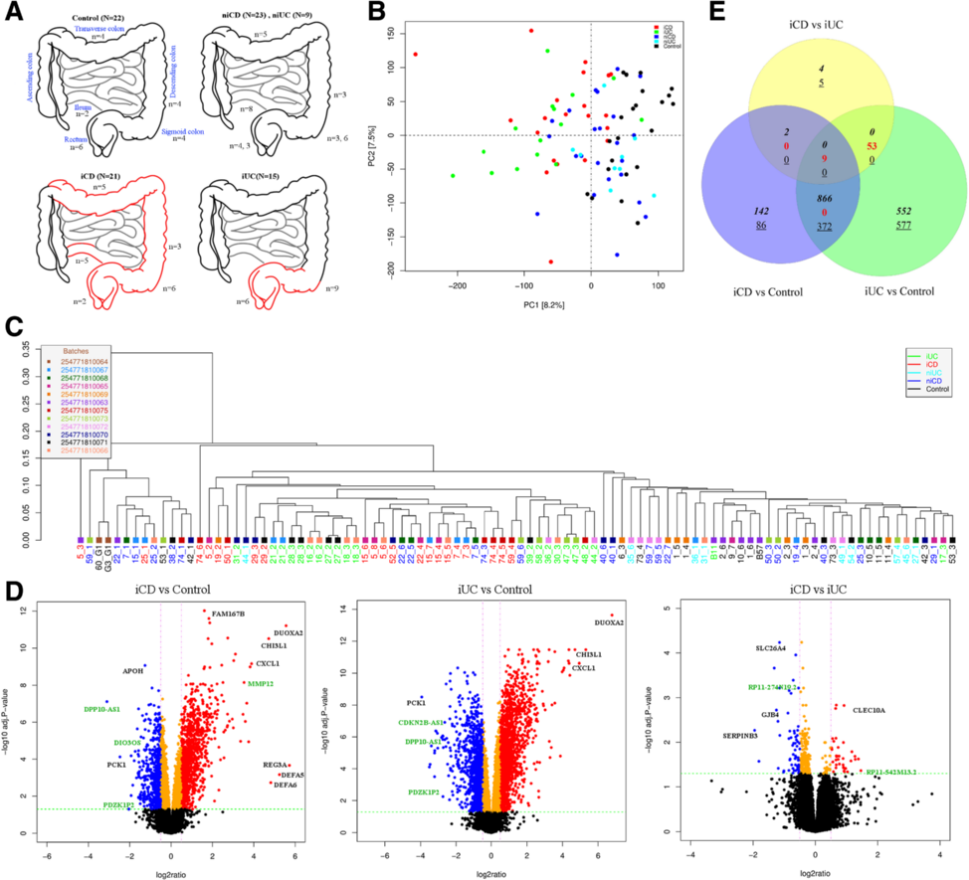
Study design and inflammatory gene signature for iCD and iUC. **(A)** Study design included 90 pinch biopsies from multiple colonic regions for both inflamed and non-inflamed mucosa (21 iCD, 23 niCD, 15 iUC, 9 niUC and 22 healthy controls samples). **(B)** Principal component analysis (PCA) separated inflamed CD (iCD) and inflamed UC (iUC) samples from non-inflamed and healthy controls. PC1 and PC2 together explained 15% of the total variation. **(C)** Unsupervised hierarchical clustering of the most dynamic probes (coefficient of variance >0.05) across the samples resulted in clustering of samples according to their clinical subgroups. **(D)** The log2 ratio and -log10 adjusted *P*-values are plotted in the form of volcano plots for iCD versus control, iUC versus control and iCD versus iUC comparisons. The probes in red, blue and orange colors represents up-regulated (FC >1.5 and adjusted *P*-value <0.05), down-regulated (FC < -1.5 and adjusted *P*-value <0.05) and significant with small fold change (FC > -1.5 and <1.5), respectively. The non-significant probes are represented in black. The selected protein-coding genes and lncRNAs labeled in black and green, respectively. **(E)** Venn diagram showing the overlap between differentially expressed genes identified in iCD versus control, iUC versus control and iCD versus iUC comparisons. The up-regulated genes are depicted in italics, down-regulated as underlined and contra-regulated in red.

An overall summary of sample information is provided in Table 1. Both, CD and UC samples were divided based on inflammation status confirmed by macroscopic and microscopic evaluations and pro-inflammatory gene signatures into inflamed (iCD, iUC) and non-inflamed (niCD, niUC) categories. The total number of samples included 21 iCD, 23 niCD, 15 iUC, 9 niUC and 22 healthy controls. In total, 90 intestinal pinch biopsies (45 individuals (13 CD, 20 UC patients and 12 healthy controls)) from multiple colonic regions were harvested from both inflamed and non-inflamed mucosa (Figure 1A). Detailed sample information, including ethnic background, disease index, previous treatment regimens and other clinical parameters, are listed in Table 2.

**Table 1:** **Overall study design and sample information**

**Diagnosis**	**Number of samples**	**Number of individuals**
**iCD**	21	13
**niCD**	23	
**iUC**	21 (15 unique)	20
**niUC**	9	
**Controls**	22	12
**Total**	96 (90 unique samples)	45

Ninety biopsy samples extracted from different colonic locations from 45 patients (CD = 13, UC = 20, controls = 12). Six samples from UC patients were used as technical replicates.

**Table 2:** **Clinical parameters**

**Number of individuals**	**CD**	**UC**	**Controls**
	**13**	**20**	**12**
**Age (median years (range))**	31 (19-59)	46 (18-68)	54 (18-77)
**Average age at diagnosis (years)**	27	33	NA
**Average years with disease (disease duration)**	8	9.3	NA
**Female/male**	6/7	13/7	8/4
**Smoking**			
Smoker (S)	8	1	4
Previous (P)	4	8	1
Never (N)	1	11	5
Not disclosed (ND)	-	-	2
**Ethnicity**			
Danish (DK)	9	19	12
European (EU)	1	-	-
Middle Eastern (ME)	3	1	-
**Number of individuals with family history of other autoimmune diseases**	1 (7%)	6 (30%)	5 (41%)
**Number of patients on medication**			NA
5-ASA	2	13	
Solumedrol	2	1	
Azathioprin	2	2	
Budesonide	1	1	
Prednisolon	1	2	
**Disease index**	HB index = 3-36	SCCAI index = 2-12	

Each column summarizes characteristics for all patients contributing samples to the corresponding sample groups. 5-ASA, 5-aminosalicylic acid; HB index, Harvey Bradshaw index; NA, not applicable; SCCAI index; Simple Clinical Colitis Activity index.

### Microarray analysis of lncRNAs and protein-coding gene expression

In the Gencode v.15 lncRNA microarray design, each lncRNA transcript is targeted by two probes covering 22,001 lncRNA transcripts corresponding to 12,963 lncRNA genes. In addition, each array contains 17,535 randomly selected protein-coding targets, of which 15,182 (unique 12,787) correspond to protein-coding genes. Six samples analyzed in duplicate, hybridized on separate chips, and used as technical replicates showed strong positive Pearson correlation (r^2^ ≥ 0.98, *P*-value <2.2e-16; Figure S1 in Additional file 2). Based on the PCA (see Methods section for details), separation of iCD and iUC samples from niCD, niUC and healthy controls were observed (Figure 1B). However, there was no apparent separation between iCD and iUC samples. The scatterplot matrices describing the first four principal components are described in Figure S2 in Additional file 2. Unsupervised hierarchical clustering of the most dynamic probes (coefficient of variance >0.05) across the samples resulted in clustering of samples according to their clinical subgroups (Figure 1C). The probes targeting lncRNAs and protein-coding genes separately also clustered samples in a similar manner (Figure S3A,B in Additional file 2).

### Differential transcriptional signature of lncRNAs and protein-coding genes in Crohn’s disease and ulcerative colitis

To define CD- and UC-specific transcriptional signatures based on intestinal inflammation, we identified differentially expressed genes using LIMMA [24] (based on a cutoff of log2 FC >1.5 (up-regulated), FC < -1.5 (down-regulated) and adjusted *P*-value <0.05 (moderated *t*-test)) in all comparisons (Table 3). The log2 ratio and -log10 adjusted *P*-values are plotted and represented as volcano plots for iCD versus control, iUC versus control and iCD versus iUC comparisons in Figure 1D. For the non-inflamed tissue comparisons (iCD versus niCD and iUC versus niUC), the volcano plots are shown in Figure S4A,B in Additional file 2, respectively.

**Table 3:** **Total number of differentially expressed genes**

	**iCD**	**niCD**	**iUC**	**niUC**
**Total differentially expressed genes**				
**Control**	1,477	73	2,429	44
**iCD**		435	73	
**iUC**				1,814
**Protein-coding genes**				
**Control**	1,039	61	1684	25
**iCD**		328	50	
**iUC**				1,215
**lncRNAs**				
**Control**	438	12	745	19
**iCD**		107	23	
**iUC**				599

Total differentially expressed genes identified in seven pairwise comparisons (iCD versus control, iUC versus control, iCD versus niCD, iUC versus niUC, niCD versus control, niUC versus control and iCD versus iUC).

Differential gene expression analysis identified the following up/down-regulated genes: 761 and 278 protein-coding genes and 254 and 184 lncRNAs in iCD versus control and 1,085 and 599 protein-coding genes and 370 and 375 lncRNAs in iUC versus control (Table 3 and Figure 1E). The top up-regulated and down-regulated lncRNAs and protein-coding genes (based on FC) for iCD versus control and iUC versus control are listed in Tables 4 and 5. Interestingly, lncRNA *RP11-731 F5.2* (whose 3′ end partly spans the start of the *IGHG2* gene) and antisense lncRNA *MMP12* were found significantly up-regulated, whereas the antisense *DPP10-AS1*, *ANRIL* (*CDKN2B-AS1)* and *DIO3OS* lncRNAs were significantly down-regulated in both iCD versus control and iUC versus control comparisons (Tables 4 and 5).

**Table 4:** **Top 10 differentially expressed lncRNA and protein-coding genes in inflamed Crohn’s disease**

**Gene name**	**Transcript**	**FC**
**Up-regulated lncRNAs**		
RP11-731 F5.2	ENST00000460164.1	14.14
MMP12	ENST00000532855.1	6.64
MMP12	ENST00000326227.5	6.52
RP11-465 L10.10	ENST00000419897.1	5.69
RP11-44 K6.2	ENST00000520185.1	3.83
FAM66D	ENST00000526690.1	3.36
LINC01272	ENST00000445003.1	3.35
RP11-44 K6.4	ENST00000522970.1	3.24
SAA2-SAA4	ENST00000524555.1	3.16
KIF9-AS1	ENST00000429315.2	3.14
**Down-regulated lncRNAs**		
DPP10-AS1	ENST00000432658.1	-8.57
PDZK1P2	ENST00000401008.2	-4.11
DIO3OS	ENST00000553575.1	-3.01
DIO3OS	ENST00000554694.1	-3.01
DIO3OS	ENST00000557532.1	-2.99
DIO3OS	ENST00000557109.1	-2.98
ANRIL (CDKN2B-AS1)	ENST00000422420.1	-2.97
ANRIL (CDKN2B-AS1)	ENST00000428597.1	-2.97
DIO3OS	ENST00000554441.1	-2.96
DIO3OS	ENST00000554735.1	-2.95
**Up-regulated protein-coding genes**		
REG3A	NM_138938	52.71
DUOXA2	NM_207581	47.26
DEFA5	NM_021010	37.73
DEFA6	NM_001926	28.33
CHI3L1	NM_001276	26.29
CXCL1	NM_001511	14.8
DMBT1	NM_007329	13.45
SAA1	NM_000331	12.67
CXCL9	NM_002416	12.07
IGHG3	ENST00000390551	11.52
**Down-regulated protein-coding genes**		
PCK1	NM_002591	-5.55
SLC26A2	NM_000112	-3.82
C10orf116	NM_006829	-3.8
GUCA2B	NM_007102	-3.61
LCN15	NM_203347	-3.43
AQP7P1	NR_002817	-3.32
TRPM6	NM_017662	-3.19
TNNC2	NM_003279	-3.1
UGT2A3	NM_024743	-2.97
ADH1C	NM_000669	-2.96

Top 10 up- and down-regulated lncRNAs and protein-coding genes in iCD versus control comparison. The log2 fold change is denoted as FC.

**Table 5:** **Top 10 differentially expressed lncRNA and protein-coding genes in inflamed ulcerative colitis**

**Gene name**	**Transcript**	**FC**
**Up-regulated lncRNAs**		
RP11-731 F5.2	ENST00000460164.1	20.64
MMP12	ENST00000532855.1	17.05
MMP12	ENST00000326227.5	16.54
RP11-465 L10.10	ENST00000419897.1	9.52
KIF9-AS1	ENST00000429315.2	5.75
FAM66D	ENST00000526690.1	5.73
SAA2-SAA4	ENST00000524555.1	5.66
CLRN1-AS1	ENST00000476886.1	4.64
RP11-1149O23.3	ENST00000517774.1	4.29
RP5-1028 K7.2	ENST00000578280.1	4.21
**Down-regulated lncRNAs**		
ANRIL (CDKN2B-AS1)	ENST00000422420.1	-8.67
ANRIL (CDKN2B-AS1)	ENST00000428597.1	-8.31
ANRIL (CDKN2B-AS1)	ENST00000585267.1	-7.06
ANRIL (CDKN2B-AS1)	ENST00000580576.1	-6.92
ANRIL (CDKN2B-AS1)	ENST00000577551.1	-6.74
ANRIL (CDKN2B-AS1)	ENST00000581051.1	-6.72
ANRIL (CDKN2B-AS1)	ENST00000582072.1	-6.68
PDZK1P2	ENST00000401008.2	-6.67
DPP10-AS1	ENST00000432658.1	-5.95
ANRIL (CDKN2B-AS1)	ENST00000421632.1	-5.78
**Up-regulated protein-coding genes**		
DUOXA2	NM_207581	109.61
CHI3L1	NM_001276	39.71
SAA1	NM_000331	30.67
CXCL1	NM_001511	25.92
MMP7	NM_002423	21.2
SLC6A14	NM_007231	20.52
IGHG3	ENST00000390551	20.14
MMP12	NM_002426	15.76
C4orf7	NM_152997	14.76
CXCL2	NM_002089	11.91
**Down-regulated protein-coding genes**		
PCK1	NM_002591	-15.24
OSTalpha	NM_152672	-11.33
ANPEP	NM_001150	-11.02
SLC26A2	NM_000112	-10.46
GBA3	NM_020973	-9.28
GUCA2A	NM_033553	-9.22
SLC3A1	NM_000341	-9.21
GUCA2B	NM_007102	-8.84
TMIGD1	NM_206832	-8.17
SLC1A7	NM_006671	-6.57

Top 10 up- and down-regulated lncRNA and protein-coding genes in iUC versus control comparison. The log2 fold change is denoted as FC.

The top differentially expressed protein-coding genes included *DUOXA2*, *CHI3L1*, *CXCL1* and *SAA1*, which were all significantly up-regulated, whereas, *PCK1*, *SLC26A2*, and *GUCA2B* were significantly down-regulated (Tables 4 and 5). In case of iCD versus controls, *REG3A* was >52-fold up-regulated (adjusted *P*-value = 2.17e-04). The top differentially expressed lncRNAs and protein-coding genes for iCD versus niCD and iUC versus niUC comparisons displayed similar expression patterns as healthy controls (Tables S1 and S2 in Additional file 1).

**Figure 2:**
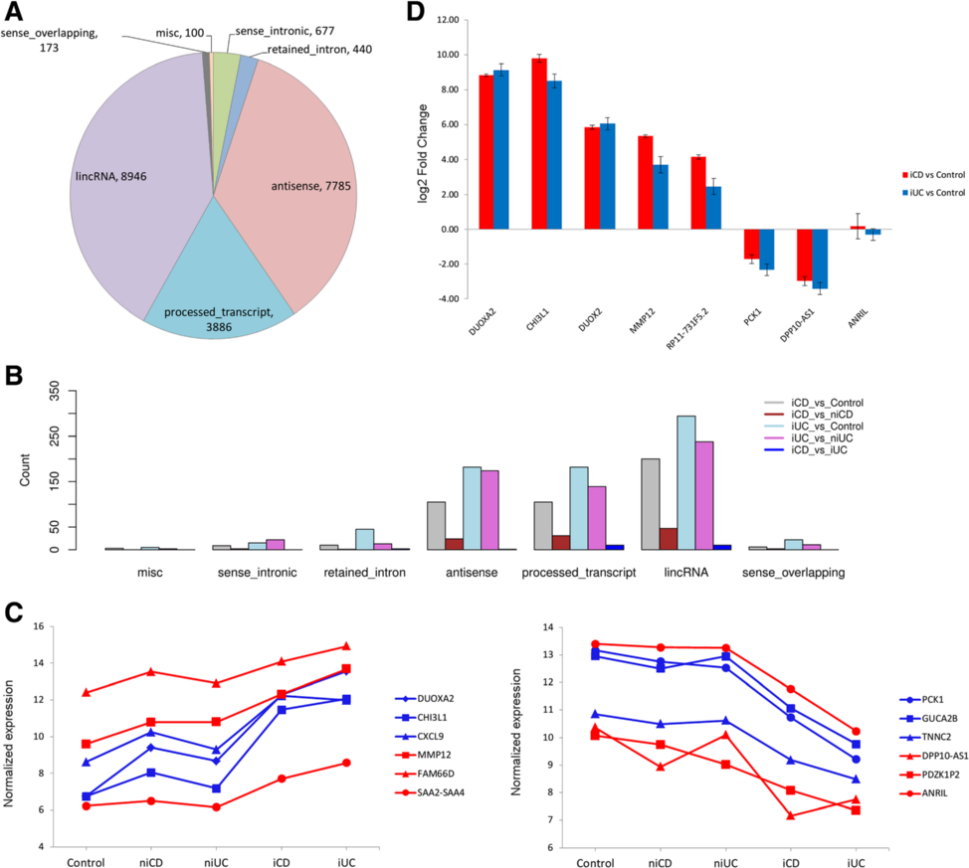
Gencode v.15 annotation of the total differentially expressed lncRNAs in IBD and microarray validation by qPCR. **(A)** The Gencode v.15 array targeted 22,007 lncRNA transcripts falling into seven major annotation classes (antisense, processed transcripts, intergenic (lincRNAs), sense overlapping, sense intronic and retained introns). Three classes (ambiguous_orf, non-coding RNAs and TEC (to be experimentally confirmed) with small number of lncRNAs were merged into a miscellaneous (misc) class for better representation. **(B)** Three major classes of differentially expressed lncRNAs identified in our study: intergenic (lincRNAs), processed transcripts and antisense lncRNAs. **(C)** Differences between the expression levels of the top three most up- and down-regulated protein-coding genes (in blue) and lncRNA genes (in red) were tested using Kruskal-Wallis test with Dunn’s multiple comparison test. The top three up-regulated protein-coding genes (*DUOXA2*, *CHI3L1* and *CXCL9*) and lncRNA genes (*MMP12*, *FAM66D* and *SAA2-SAA4*) showed increasing signal intensity from control group to inflamed CD and UC groups based on averaged gene expression levels (*P*-value <0.001). The top three down-regulated protein-coding genes (*PCK1*, *GUCA2B* and *TNNC2*) and lncRNA genes (*DPP10-AS1*, *PDZK1P2* and *ANRIL*) showed decreasing signal intensity across the clinical subgroups from iCD and iUC to controls (*P*-value <0.001). **(D)** A total of eight genes were selected for real-time PCR validation of the microarray data in iCD versus control (red) and iUC versus control (blue). The log2 fold change is plotted on the y axis.

On comparing niCD versus control and niUC versus control, only a small number of up/down-regulated genes (61 and 25 and 8 and 17 protein-coding, 12 and 19 and 9 and 10 lncRNA) were identified for niCD and niUC, respectively. Nearly all of the differentially expressed genes in niCD versus control were also present in the iCD versus control comparison with the exception of the protein-coding gene *CRYBB2* (FC = -1.5; Table S3 in Additional file 1). Conversely, for niUC versus control, most (15 out of 17) of the up-regulated genes, including four small nucleolar RNAs (snoRNAs: *SNORD97*, *SNORA28*, *SNORA53*, and *SNORA74A*) and the down-regulated genes (*MAST3*, *CPT1B*, *LOC338799*, *EXOC3L4*, and *MAPK8IP3*) were specifically found in niUC only (Table S4 in Additional file 1). Importantly, in the iCD versus iUC comparison, 18 and 32 protein-coding genes and 13 and 10 lncRNAs were significantly found to be up/down-regulated, respectively. The top up/down-regulated lncRNAs and protein-coding genes for iCD versus iUC are shown in Table 6. Annotations for the Gencode v.15 [38] microarray features for lncRNAs are summarized in Figure 2A. Most of the differentially expressed lncRNAs identified in our analysis belonged to three main classes: antisense, processed transcripts and intergenic lincRNAs (Figure 2B), as described in the following section.

**Table 6:** **Top 10 differentially expressed genes in inflamed Crohn’s disease versus inflamed ulcerative colitis**

**Gene name**	**Transcript**	**FC**
**Up-regulated lncRNAs**		
FLJ42969	ENST00000514926.1	2.6
AC007182.6	ENST00000455232.1	2.42
RP11-542 M13.2	ENST00000599411.1	2.04
RP11-399 F4.4	ENST00000453998.1	1.87
FAM95B1	ENST00000455995.1	1.87
RP3-395 M20.8	ENST00000432521.2	1.69
RP3-395 M20.8	ENST00000448624.2	1.65
OPLAH	ENST00000426825.1	1.61
OPLAH	ENST00000534424.1	1.61
SPPL2B	ENST00000592738.1	1.59
**Down-regulated lncRNAs**		
AL928742.12	ENST00000412518.1	-2.01
RP11-444D3.1	ENST00000540811.1	-1.84
AL928742.12	ENST00000427543.1	-1.8
FAM25D	ENST00000426412.2	-1.69
RP11-274 N19.2	ENST00000515643.1	-1.64
RP11-838 N2.4	ENST00000579007.1	-1.59
RP11-279 F6.3	ENST00000558941.1	-1.57
RP11-279 F6.3	ENST00000559212.1	-1.55
LINC00524	ENST00000555860.1	-1.54
VAV3-AS1	ENST00000438318.1	-1.52
**Up-regulated protein-coding genes**		
C8G	NM_000606	2.75
SLC25A34	NM_207348	2.43
UGT1A6	NM_001072	2.25
LRRC66	NM_001024611	2.20
EXOC3L4	NM_001077594	1.95
ANO7	NM_001001891	1.95
GLYCTK	NM_145262	1.90
CLEC10A	NM_182906	1.89
FAM95B1	NR_026759	1.89
LPIN3	NM_022896	1.87
**Down-regulated protein-coding genes**		
SERPINB3	NM_006919	-3.87
SLC6A14	NM_007231	-3.51
GAL	NM_015973	-2.50
GJB4	NM_153212	-2.38
IGHV1-58	ENST00000390628	-2.29
CRYM	NM_001888	-2.28
SLC26A4	NM_000441	-2.22
DEFB103B	NM_018661	-2.22
LAMC2	NM_005562	-2.20
TUSC3	NM_178234	-2.02

Top 10 up- and down-regulated lncRNAs and protein-coding genes in iCD versus iUC. The log2 fold change is denoted as FC.

Additionally, we also tested the differences between the clinical subgroups for the top differentially expressed protein-coding and lncRNA genes. The top three up-regulated protein-coding genes (*DUOXA2*, *CHI3L1* and *CXCL9*) and lncRNA genes (*MMP12*, *FAM66D* and *SAA2-SAA4*) showed increasing signal intensity based on the averaged gene expression levels across the spectrum of clinical subgroups from control to iCD and iUC (*P*-value < 0.001; Figure 2C). For the top three down-regulated protein-coding genes (*PCK1*, *GUCA2B* and *TNNC2*) and lncRNA genes (*DPP10-AS1*, *PDZK1P2* and *ANRIL*), we observed decreasing signal intensity across the clinical subgroups from iCD and iUC to controls (*P*-value <0.001; Figure 2C). Importantly, eight major isoforms (out of a total of 17 annotated isoforms) of *ANRIL* were found to be down-regulated in iCD and iUC compared with controls and non-inflamed tissues in our data (Tables 4 and 5). *ANRIL* was -2.97-fold and -2.72-fold down-regulated in iCD versus control and iCD versus niCD comparisons, and -8.31-fold and -7.98-fold down-regulated in iUC versus control and iUC versus niUC comparisons, respectively.

Furthermore, for the validation of microarray results by qPCR, we selected eight top differentially expressed genes (based on FC) common between iCD versus control and iUC versus control (up-regulated: *DUOXA2*, *CHI3L1*, *DUOX2*, *MMP12*, *RP11-731 F5.2*; down-regulated: *PCK1*, *DPP10-AS1*, *ANRIL*). The qPCR analysis confirmed the microarray expression results with respect to the fold change values (Table S5 in Additional file 1). We also performed qPCR analysis for *DUOX2*, although it was not probed on our microarray, but it has been implicated along with its maturation factor, *DUOXA2*, in IBD pathogenesis (see Discussion). Both *DUOXA2* and *DUOX2* were found to be significantly up-regulated in iCD versus control (FC = 8.83 and 5.85, respectively) and iUC versus control (FC = 9.14 and 6.05, respectively) (Figure 2D). For the remaining four comparisons, we also tested five differentially expressed genes by qPCR validation (Table S6 and S7 in Additional file 1).

### Overlap of differentially expressed genes in inflamed Crohn’s disease and inflamed ulcerative colitis

**Figure 3:**
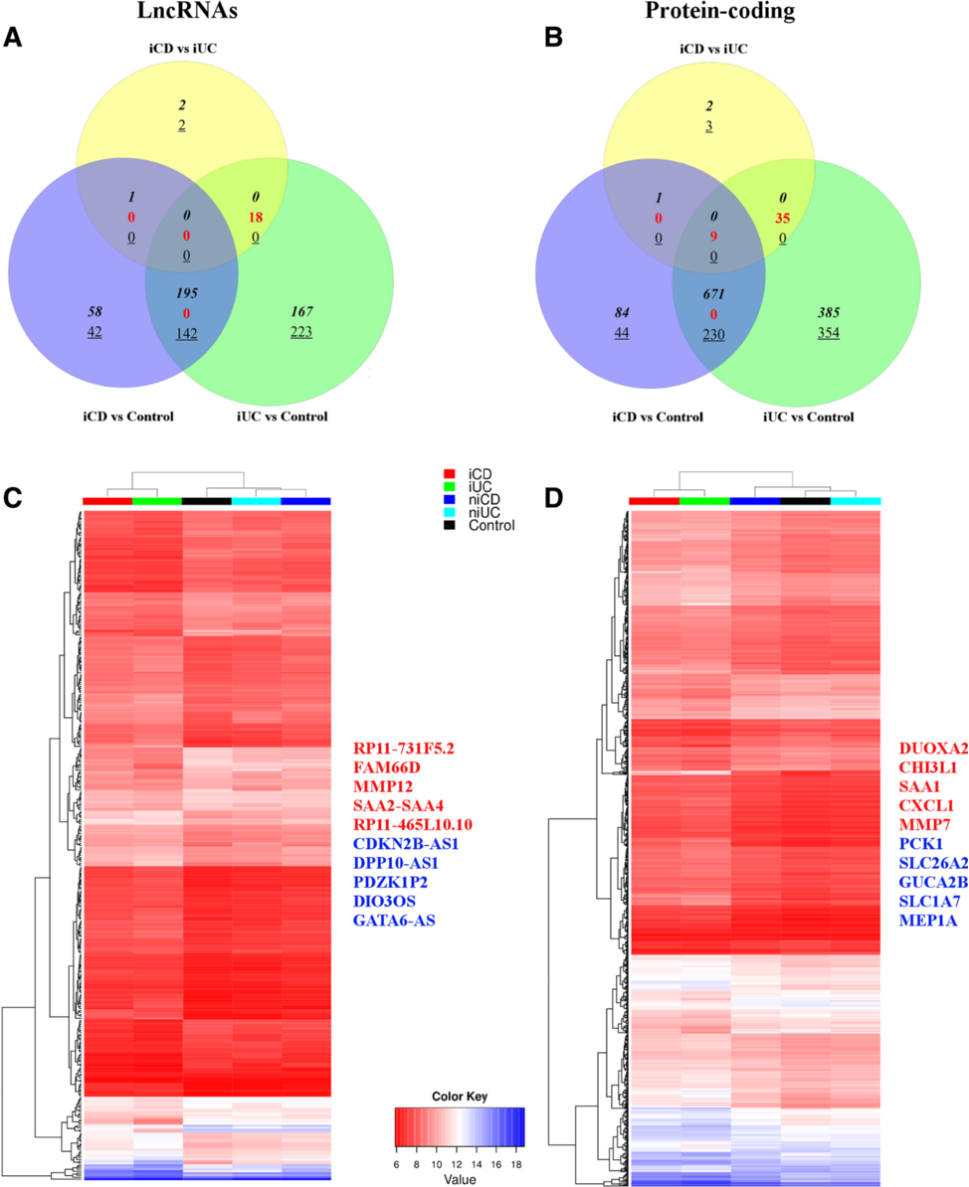
Overlap of differentially expressed lncRNAs and protein-coding genes between iCD and iUC. **(A,B)** Venn diagrams show an overlap of 337 lncRNAs (A) and 901 protein-coding genes (B) that were differentially expressed (FC >1.5, adjusted *P*-value <0.05) between patients with iCD and iUC compared with healthy controls. We observed contra-regulated genes between iCD/iUC versus control comparisons compared with the iCD versus iUC comparison. The up-regulated genes are depicted in italics, down-regulated as underlined and contra-regulated in red. **(C,D)** Heat maps of average normalized gene expression for the overlapping 337 lncRNAs (C) and 901 protein-coding genes (D) between iCD and iUC in the five clinical subgroups (iCD, niCD, iUC, niUC and controls) are displayed. Selected up-regulated and down-regulated genes are listed.

A Venn diagram illustrating the relationship between lncRNAs and protein-coding genes differentially expressed in iCD and iUC is shown in Figure 3. In total, 337 differentially expressed lncRNAs were identified as common between iCD and iUC with 100 unique lncRNAs for iCD and 400 unique lncRNAs for iUC (compared with the healthy controls; Figure 3A). For the protein-coding genes, 901 differentially expressed genes were found to be common between iCD and iUC with 128 unique for iCD and 739 unique for iUC (Figure 3B). Conversely, in the iCD versus iUC comparison, 19 out of 23 and 45 out of 50 differentially expressed lncRNAs and protein-coding genes, respectively, overlapped with iCD versus control and iUC versus control.

The unsupervised hierarchical clustering showed that both inflamed groups (iCD and iUC) cluster together, in contrast to the non-inflamed groups (niCD and niUC), which clustered with healthy controls. The normalized gene expression values from the above-mentioned 337 lncRNAs and 901 protein-coding genes common to both iCD and iUC conditions were averaged for each of the five clinical subgroups and are visualized in a heat map in Figure 3C,D. The expression patterns for the specific up-regulated and down-regulated genes showed increasing or decreasing signal intensity across the clinical subgroups (from iCD, iUC, niCD, niUC and healthy controls). Collectively, these overlapping differentially expressed genes between iCD versus control and iUC versus control define a distinct inflammatory iCD/iUC gene expression signature. Importantly, this inflammatory gene signature included the key drivers of the innate and adaptive immune responses (for example, *DUOXA2* and *CXCL1*).

### Comparison of expression levels of top differentially expressed genes in patients and healthy controls

**Figure 4:**
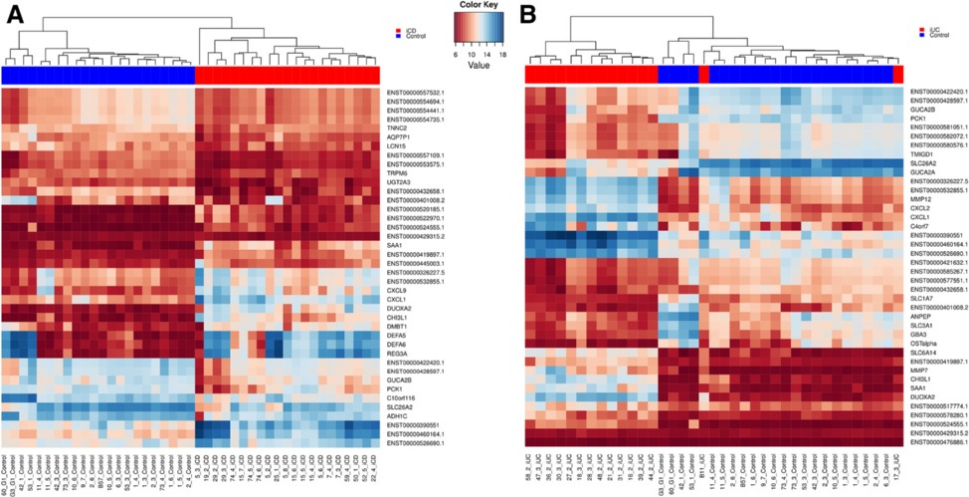
Comparison of expression levels of the top 40 differentially expressed lncRNAs and protein-coding genes. **(A,B)** Unsupervised hierarchical clustering of samples (patients in red, controls in blue) based on normalized expression values from the top 40 up- and down-regulated lncRNAs and protein-coding genes for iCD versus control (A) and iUC versus control (B). The log2 normalized expression values are shown in the color key. A clear separation between the diseased and control groups is visible in the case of the iCD versus control comparison.

To stratify iCD and iUC samples from the healthy controls, we also compared the expression profiles of the top 20 up/down-regulated lncRNAs and the top 20 up/down-regulated protein-coding genes (based on FC) through unsupervised hierarchical clustering. An expression map of these top 40 differentially expressed genes displayed a clear separation of the patients from the control groups (Figure 4A,B), except for the two iUC samples B11 and 17_3, which were misclassified in the clustering. The magnitude of log2 intensity signal for these top differentially expressed genes displayed in Figure 4 was >6 in both iCD and iUC. Interestingly, in the case of the iCD versus iUC comparison, clustering was unable to distinguish between iCD and iUC patients (Figure S5 in Additional file 2). In addition to the top candidates, we also compared the expression profiles of all differentially expressed lncRNAs and protein-coding genes, and observed similar results as described above (Figure S6A,B in Additional file 2).

### Inflammatory response and antimicrobial peptide genes are dysregulated in inflamed Crohn’s disease and inflamed ulcerative colitis

**Figure 5:**
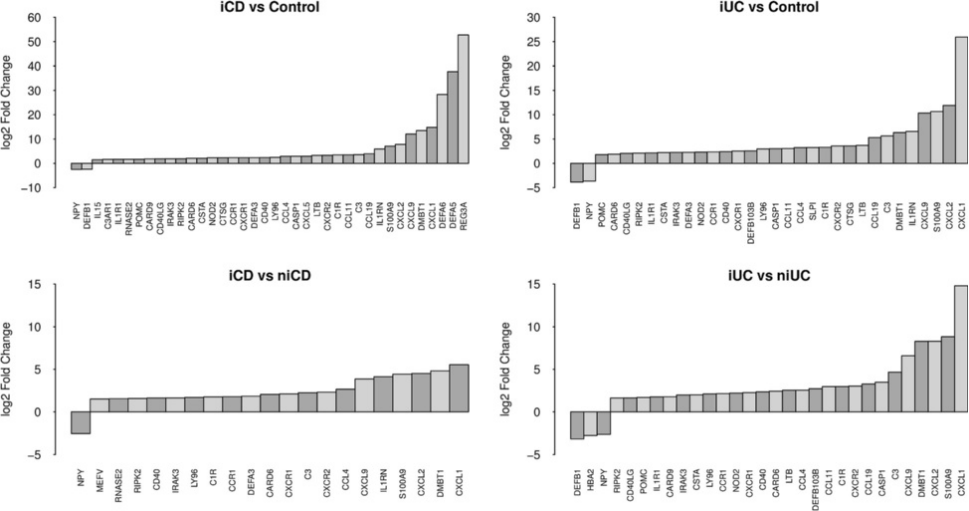
Differentially expressed protein-coding genes involved in antimicrobial and autoimmune responses. Key genes involved autoimmune and inflammatory immune responses and AMPs were found to be dysregulated in both iCD and iUC compared with healthy controls as well as non-inflamed tissues. The log2 FC values are plotted on the y-axis.

Antimicrobial peptides (AMPs) play an important role in protecting the host intestinal mucosa against microorganisms and AMP dysregulation has been associated with IBD pathogenesis (see Discussion for details). Therefore, we investigated whether there were differences in the expression of genes involved in the inflammatory response and AMP production between the different clinical subgroups. Our analysis identified key genes associated with the inflammatory response, including the pro-inflammatory chemokines and cytokines. *CCL11*, *CCL19*, *CCL4* and *CXCL9* were significantly up-regulated in both iCD versus control and iUC versus control. In addition, we also found key antimicrobial response genes to be significantly up-regulated in iCD and iUC compared with healthy controls (Figure 5). *REG3A*, *DEFA5* and *DEFA6* were >30-fold up-regulated only in iCD versus control. The chemokines *CXCL1* and *CXCL2* were >15- and >25-fold up-regulated in both iCD versus control and iUC versus control, respectively. *CXCL5*, *IL15* and *C3AR1* were specifically up-regulated in iCD versus control (Figure 5). Notably, the *NOD2* gene was >2-fold up-regulated in iCD versus control, iUC versus control and iCD versus niCD comparisons. *DEFB1* and *NPY* were the only AMP genes that were significantly down-regulated in both iUC and iCD.

### Differentially expressed genes in inflamed Crohn’s disease and inflamed ulcerative colitis are enriched within inflammatory bowel disease loci

**Figure 6:**
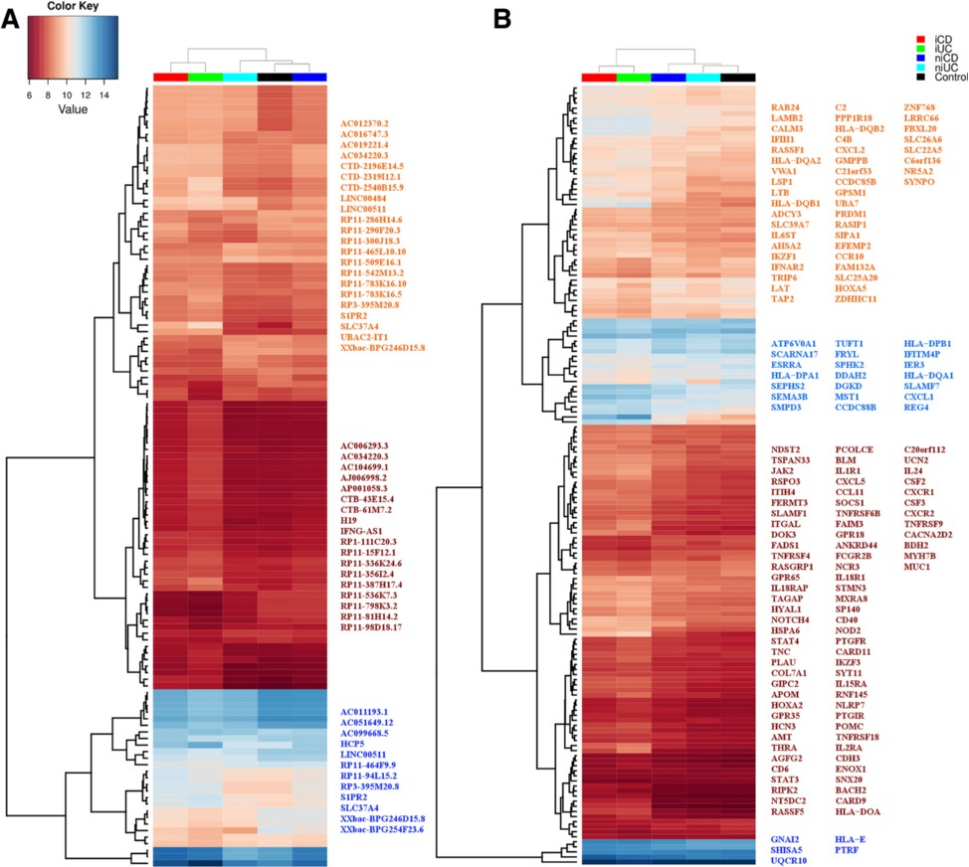
Averaged gene expression for differentially expressed IBD loci-associated lncRNAs and protein-coding genes. **(A,B)** Unsupervised hierarchical clustering of averaged and normalized expression values for 96 differentially expressed IBD loci-associated lncRNAs (A) and 154 protein-coding genes (B) in different clinical subgroups. The range for the expression values is shown in the color scale.

Since most disease-associated susceptibility SNPs map to the non-coding regions in the genome, we looked for the presence of known IBD-associated SNPs (total of 233 SNPs) within the Gencode v.15 annotated lncRNAs. Interestingly, 29 IBD risk variants intersected 37 lncRNAs, of which only the *IFNG-AS1* antisense lincRNA (*ENST00000536914.1*) harboring the UC susceptibility SNP rs7134599 was found to be differentially expressed in our study. *IFNG-ASI* was up-regulated in iUC versus control (FC = 1.54) and iUC versus niUC (FC = 1.52). Furthermore, we identified IBD loci-associated lncRNAs and protein-coding genes by intersecting the IBD susceptibility loci, which was defined as a 500 kb long genomic region with the IBD risk variant in the middle. In total, 1,040 IBD loci-associated lncRNAs were identified, out of which 96 lncRNAs were found to be differentially expressed (Table S8 in Additional file 1). These differentially expressed lncRNAs co-localized with 57 IBD risk variants (within a 500 kb locus), and were found to be enriched within IBD loci (*P*-value <0.0001, Pearson’s Chi-squared test). In the case of protein-coding genes, 681 genes were found to be associated with IBD loci, out of which 154 were differentially expressed and enriched within IBD loci (*P*-value <0.0001, Pearson’s Chi-squared test). Unsupervised hierarchical clustering of averaged and normalized gene expression values of 96 and 154 differentially expressed IBD loci-enriched lncRNAs and protein-coding genes, respectively, enabled independent stratification of disease from the controls and further distinguished inflamed from non-inflamed conditions in both CD and UC (Figure 6).

### Regulatory IBD-associated SNPs co-localize with differentially expressed IBD loci-associated lncRNAs

Next, we asked whether active regulatory regions within the IBD loci overlap with the differentially expressed IBD loci-associated lncRNAs. IBD-associated SNPs overlapping active regulatory elements in intestinal epithelium were retrieved from the Mokry *et al*. study [7]. In their study, the active regions overlapping IBD-associated SNPs were identified based on H3K27ac chromatin immunoprecipitation and sequencing (ChIP-Seq). Out of 96 differentially expressed IBD loci-associated lncRNAs, 68 were found to be associated with 24 IBD loci SNPs co-localizing with the active regulatory elements in intestinal epithelium and immune cells (Table S8 in Additional file 1). These overlapping IBD loci-associated active regulatory elements have been reported to frequently co-localize with the known transcription factor binding motifs [7]. A number of IBD-associated SNPs potentially affect the binding affinity of transcriptional factors, and thus perturb the gene expression. Additionally, IBD-associated risk variants also act as expression quantitative trait loci (eQTLs) signals for a number of genes (Table S8 in Additional file 1). For example, IBD-associated risk variant rs10797432, located within the IBD loci-associated lncRNA *RP3-395 M20.8* (*ENSG00000238164*), alters the binding motifs for TFAP2A and CTCF. Furthermore, it is also known to act as a *cis*-eQTL for *MMEL1* in monocytes. The regulatory IBD-associated SNP rs1569723, located within the IBD loci-associated lncRNA *RP11-465 L10.10* (*ENSG00000204044*), acts as a *cis*-eQTL for *CD40* in monocytes. Also, SNP rs12946510, associated with lncRNAs *RP11-387H17.4* (*ENSG00000264968*) and *RP11-94 L15.2* (*ENSG00000264198*), is known to perturb the binding sites for transcription factors FOXO1, ELF3, and SRF. In addition, this SNP also acts as a *cis*-eQTL for the pseudogene *KRT222P*, transcriptional co-activator complex component *MED24*, transcription factor *NR1D1*, and *ORMDL3* in lymphoblastoid cell lines. In the case of the antisense lncRNA *CTD-2196E14.5* (*ENSG00000261266*), the associated SNP rs7404095 acts as a *cis*-eQTL for *PRKCB* in lymphoblastoid cell lines and *PRKCB1* in monocytes. Moreover, SNP rs734999, associated with lncRNA *RP3-395 M20.8* (*ENSG00000238164*), acts as a *cis*-eQTL for *TNFRSF14* in lymphoblastoid cell lines.

### *Cis*-acting correlation of expression between differentially expressed inflammatory bowel disease loci-associated lncRNAs and protein-coding genes

**Figure 7:**
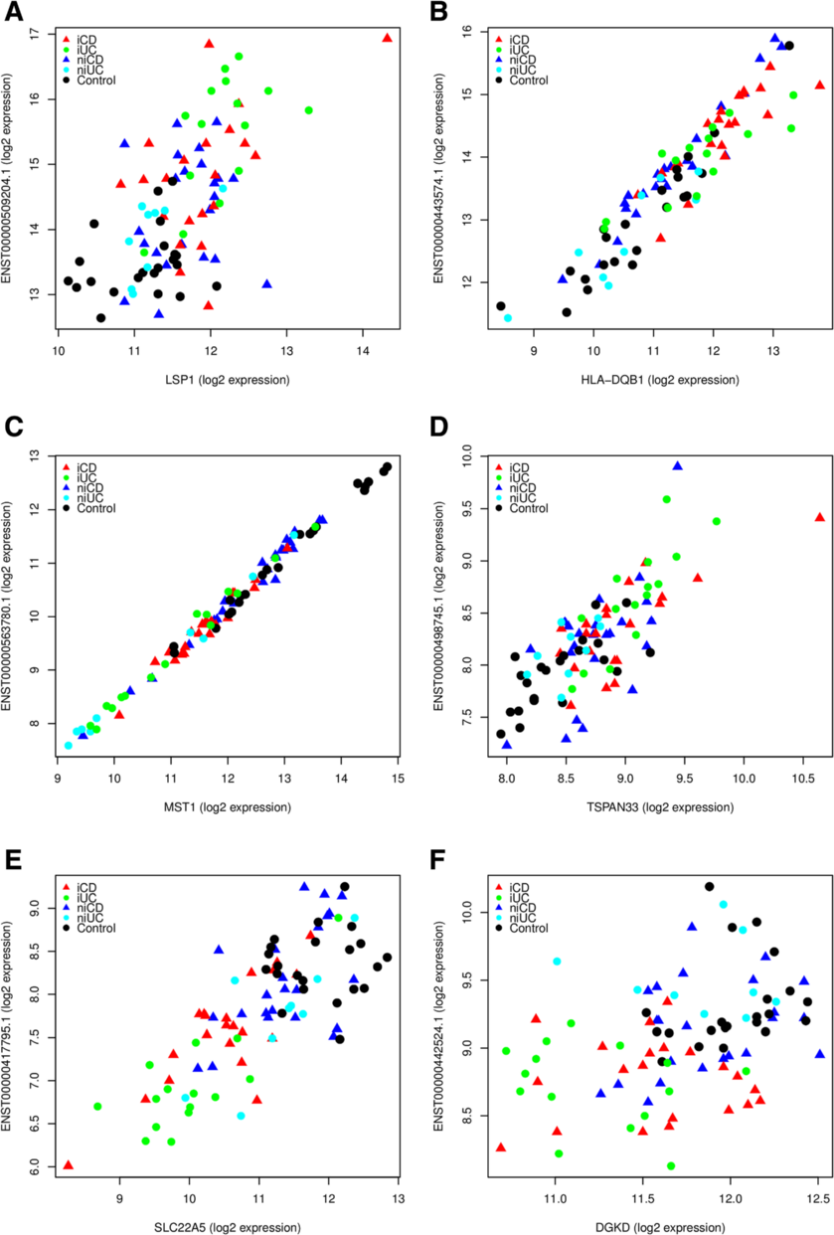
Correlations of expression for *cis-*neighboring pairs of IBD loci-associated differentially expressed lncRNAs and protein-coding genes. **(A-F)** Overall positive correlations between overlapping protein-coding and lncRNA gene-pairs: *LSP1* and *ENST00000509204.1* (A); *HLA-DQB1* and *ENST00000443574.1* (B); *MST1* and *ENST00000563780.1* (C); *TSPAN33* and *ENST00000498745.1* (D); *SLC22A5* and *ENST00000417795.1* (E); *DGRD* and *ENST00000442524.1* (F). An extremely positive (r^2^ ≥ 0.99, *P*-value <2.2e-16) correlation was observed for *MST1* and its intersecting lncRNA *ENST00000563780.1*, which is associated with IBD risk variants rs9822268 and rs3197999. Protein-coding expression is plotted on the x-axis, and lncRNA expression is shown on the y-axis. Each point represents a biopsy sample from different clinical subgroups.

We computed pairwise Pearson correlations in order to explore the possible co-expression patterns between IBD loci-associated differentially expressed lncRNAs and protein-coding genes. Pairwise correlations of expression involving neighboring lncRNAs and protein-coding genes associated with each IBD-associated SNP (500 kb loci with the SNP in the middle) were computed. We found positive (r^2^ ≥ 0.5) and extremely positive (r^2^ ≥ 0.9) correlations between the overlapping as well as *cis*-neighboring differentially expressed IBD loci-associated lncRNA-protein-coding gene pairs (*P*-value <0.05). The pairwise correlations for six intersecting IBD loci-associated lncRNA-protein-coding gene pairs - *LSP1* and *ENST00000509204.1* (rs907611), *HLA-DQB1* and *ENST00000443574.1* (rs9268853, rs6927022), *MST1* and *ENST00000563780.1* (rs9822268 and rs3197999), *TSPAN33* and *ENST00000498745.1* (rs4728142), *SLC22A5* and *ENST00000417795.1* (rs2188962, rs12521868), *DGRD* and *ENST00000442524.1* (rs12994997, rs3792109) - are plotted in Figure 7. Interestingly, lncRNA *ENST00000563780.1* and *MST1* protein-coding gene exhibited extremely positive correlation (r^2^ ≥ 0.99; Figure 7). Enrichment for positive correlations has been reported for the lncRNAs intersecting protein-coding genes in an antisense orientation [35]. Indeed, we also observed strong positive correlation (r^2^ ≥ 0.7) for the intersecting antisense lncRNA *ENST00000417795* and the protein-coding gene *SLC22A5*.

### Functional annotation of differentially expressed lncRNAs

The functional annotations of lncRNAs have mostly been based on the nearest-neighbor approach, that is, ‘guilt-by-association’ analyses - for example, Cabili *et al*. [39]. We therefore analyzed the GO terms of genes that overlap with or are neighbors of the differentially expressed lncRNAs. We identified 516 nearest protein-coding neighbors within a span of <10 kb covering 610 differentially expressed lncRNAs. In addition, we also included 712 neighboring protein-coding genes for the 57 IBD risk variants (associated with 96 differentially expressed IBD loci-associated lncRNAs) based on a 1 Mb locus size for each variant. The most significant over-represented GO terms in the biological process category included antigen processing and presentation (*P*-value 7.39e-08), immune system process (*P*-value 2.5e-05) and natural killer cell activation (*P*-value 9.6e-05) (Table S9 in Additional file 1). In the cellular component category, we found enrichment for the MHC protein complex (*P*-value 5.95e-09). Furthermore, we also observed enrichment for over-represented GO terms in the molecular function category, which included protein binding, receptor binding and cytokine activity.

### Cross-validation of differentially expressed genes by SVM

**Figure 8:**
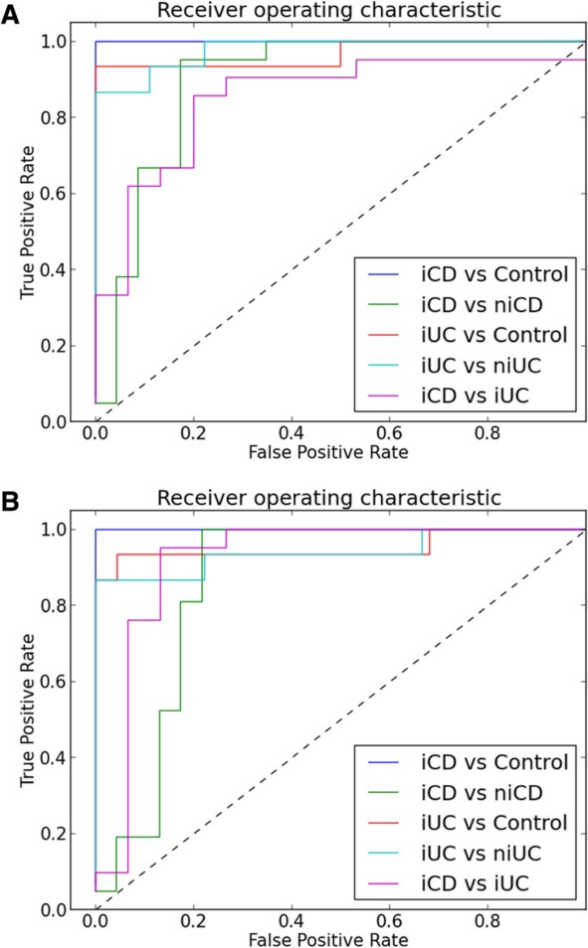
Receiver operating characteristic curve analysis of differentially expressed lncRNAs and protein-coding genes. **(A)** lncRNAs and **(B)** protein-coding genes for five comparisons.

SVM [33] was used for classifying IBD cases from controls and for cross-validating differentially expressed genes identified by LIMMA. The best SVM classifier performance was obtained from differentially expressed lncRNAs identified in the iCD versus control followed by iUC versus control comparison (see Methods for details). The classifier distinguished iCD and iUC from controls with 100% and 94.6% accuracy, 100% and 100% specificity and 100% and 86.7% sensitivity, respectively. In addition, the classifier was also able to distinguish iCD and iUC from niCD and niUC samples with an accuracy of 86.4% and 91.7%, specificity of 78.3% and 88.9% and sensitivity of 95.2% and 83.3%, respectively. For the iCD versus iUC comparison, the accuracy of the classifier was 77.8%, with 60.0% specificity and 90.4% sensitivity (Figure 8A). For the differentially expressed protein-coding genes, the classifier achieved an accuracy of 100% and 94.6%, with 100% and 100% specificity, and 100% and 86.7% sensitivity, in discriminating iCD and iUC from controls, respectively (Figure 8B). Similar to the above described observations, the classifier also allowed iCD and iUC to be distinguished from niCD and niUC samples with an accuracy of 81.8% and 83.3%, specificity of 78.2% and 77.8% and sensitivity of 85.7% and 86.7%, respectively. For the iCD versus iUC comparison, the accuracy of the classifier was 88.9%, with 80.0% specificity and 95.2% sensitivity (Figure 8B). Furthermore, our classifier achieved a similar performance when using combined differentially expressed protein-coding and lncRNA genes or only protein-coding genes (data not shown). The effect of clinical parameters (Table 2; Figure S7 in Additional file 2) on disease (iCD or iUC) was described by the following linear function:

$$ y = 0.511 + \left(-0.212* age\right) + \left(-0.114* sex\right) + \left(-0.339* smoking\right) + \left(1.185* disease\  score\right) + \left(-0.058* biopsy\  location\right) $$

Using t-statistics, *P*-values for linear regression coefficients for age, sex, smoking, disease index and biopsy location were 1.40e-01, 2.29e-01, 2.98e-03, 5.02e-07 and 6.43e-01, respectively. Our analysis indicated that disease index had the strongest effect on defining iCD and iUC phenotype, followed by smoking, sex and age (Figures S7 and S8 in Additional file 2). Differentially expressed genes identified by LIMMA were verified by SVM-RFE [35], which revealed a robust concordance rate in terms of total number of overlapping differentially expressed genes identified by the two methods (Figure S9 in Additional file 2). In both the iCD versus control and iUC versus control comparisons, an overlap of about 66% was observed. To control for any input bias, a randomized lncRNA gene list of the same size as the differentially expressed lncRNAs was also used in this analysis (Figure S8 in Additional file 2).

### Impact of clinical parameters on disease diagnosis

We next investigated the impact of the clinical parameters for disease diagnosis, and whether expression profiles of differentially expressed genes are also correlated with other clinical parameters. The applied strategies were linear regression model and WGCNA [36]. The regression analysis showed a strong impact of the disease index (Harvey-Bradshaw Index for CD and Simple Clinical Colitis Activity Index for UC; *P*-value <10e-6, *t*-test), which by definition is positively correlated with the severity of the disease, and a significant impact of smoking (*P*-value <0.05, *t*-test), which was, however, 3.5 times lower than the disease index and lower than the error rate. However, biopsy location did not show any significant effect on the severity of the disease. In agreement, the network analysis identified 10,435 genes significantly correlated with the disease index (*P*-value <0.05, *t*-test), which is a clinical parameter with most related gene expression profiles. However, only 509 of these genes were differentially expressed between disease and control. Conversely, the expression profile of 1,006 differentially expressed genes significantly associated with age (*P*-value <0.05, *t*-test; Table S10 in Additional file 1). These results suggest that even though the sample diagnosis for disease is only partly related to other clinical parameters, especially disease index and smoking, many differentially expressed genes in iCD and iUC also reflect an impact of the patient’s age. The average gene significance measures for all genes in a given module are summarized in Table S10 in Additional file 1.

Overall, the network analysis identified three large co-expression modules enriched for differentially expressed genes between iCD/iUC and control (*P*-value <10e-100, Pearson’s Chi-squared test; Figures S10 and S11 in Additional file 2). The three modules comprised 2,054 out of 2,737 differentially expressed genes. The gene network of the ‘brown’ module was found to be enriched for immune and pro-inflammatory responses (Table S11 in Additional file 1), the ‘green’ and ‘red’ modules were driven by genes involved in small molecule trans-membrane transport, and anionic and cationic transport (Tables S12 and S13 in Additional file 1). GO analysis was also performed for the randomized gene sets of the same module sizes. None of the randomized modules had significant GO terms.

## Discussion

The present study was intended to explore the transcriptomic landscape of lncRNAs in IBD, with particular focus on CD and UC. To explore the transcriptomic profiles of CD and UC patients, colonic pinch biopsies were analyzed using gene expression microarrays. Our results revealed widespread dysregulation of lncRNA and protein-coding gene expression in both CD and UC. It is noteworthy that although our main focus was transcriptome analysis of lncRNAs, we also profiled a significant number of protein-coding genes (approximately 12,000; see Methods). The Gencode v.15 lncRNA microarray has been extensively used and the levels of both mRNAs and lncRNAs are comparable and show strong correlations (ranging from 0.62 to 0.75) with results obtained from RNA sequencing (RNAseq) [38]. These correlations are also comparable with the previous lncRNA microarray versions [35]. The Gencode v.15 lncRNA microarray has been designed to capture both poly(A) and non-poly(A) transcripts (out of a total 22,007 lncRNA transcripts targeted by the microarray, 9,273 lncRNA transcripts are polyadenylated). In recent years, many studies have been conducted to profile lncRNAs using RNAseq; however, this is expensive and time consuming because of the requirement of doing deep sequencing, particularly for lncRNAs, which are expressed at relatively lower levels than protein-coding genes [38]. It has also been reported that microarrays are more sensitive to detect whether a lncRNA is expressed or not compared with RNAseq [40].

SVM-based classifiers have been previously used to cross-validate the circulating microRNA-based biomarker panels in UC [13]. We also verified the robustness of the differentially expressed genes by SVM and the predictive capability of these genes to discriminate CD and UC was tested using SVM-RFE-based classifiers. The Harvey-Bradshaw Index and Simple Clinical Colitis Activity Index are symptom-based indices used to assess the disease activity in CD and UC, respectively. Among various clinical parameters tested, we found strong influence of the disease index, followed by smoking, age and sex, on iCD and iUC phenotypes. Smoking is known to have deleterious effects in CD while it has been found to be protective against UC [41]. Furthermore, smoking has also been shown to influence the colonic gene expression profile in CD [42]. Additionally, based on linear regression and WGCNA, we did not find any significant effect of biopsy location on overall gene expression. However, regional variation in gene expression along the colonic mucosa has been reported to influence expression profiling studies in IBD [43,44]. These modest regional variations are more pronounced in healthy controls and un-inflamed biopsies and largely remain masked when comparing inflamed biopsies [44]. On the contrary, other studies suggest no such gene expression differences due to regional variation [17,45]. These reports highlight the importance and impact of various confounding factors like smoking, sex, and biopsy locations among many other clinically relevant parameters in gene expression analysis in IBD.

Our analysis identified common expression patterns between the lncRNAs and protein-coding genes in iCD and iUC as confirmed by unsupervised hierarchical clustering (Figure 3). A distinctive inflammatory (iCD/iUC) gene expression signature included the key drivers of the innate and adaptive immune responses (chemokines, cytokines and defensins) - for example, *DUOXA2* (dual oxidase maturation factor 2), *CXCL1* (chemokine (C-X-C motif) ligand 1), *CXCL9* (chemokine (C-X-C motif) ligand 9) - and also included a significant number of lncRNAs. Expression levels of both *DUOX2* and *DUOXA2* have been reported to be up-regulated in association with iUC, and in UC-associated colorectal dysplasia and colorectal cancer and are involved specifically in inflammation and regulated on a crypt-by-crypt basis in UC [46]. We also observed a global up-regulation of *DUOXA2* in iCD and iUC compared with both non-inflamed and healthy controls. Both *DUOX2* and its maturation factor *DUOXA2* are part of the *NADPH* oxidase family of enzymes involved in release of hydrogen peroxide (H_2_O_2_) [47]. These enzymes are essential components of evolutionarily conserved mechanisms through which organisms are known to defend themselves against bacterial, viral, or parasitic infections, yet allowing tolerance of commensals [48,49] Suppression of *DUOX2*-generated H_2_O_2_ production by mesalazine (5-aminosalicylic acid) has been demonstrated to reduce reactive oxygen species-induced genetic lesions and thereby lowering the risk of UC-associated colorectal dysplasia and colorectal cancer [46].

Our results revealed significant down-regulation of the lncRNA *ANRIL* (antisense non-coding RNA in the INK4 locus) in both iCD (FC < -2.7, *P*-value <0.05) and iUC (FC < -7.9, *P*-value <0.05) compared with non-inflamed and healthy controls. *ANRIL*, encoded on the chromosome 9p2.3 region, is a known hotspot for disease-associated SNPs [50]. *ANRIL* has emerged as an important regulatory molecule mediating human disease at various levels and cellular settings. Nevertheless, the role of *ANRIL* has not yet been described specifically in the context of IBD pathology. *ANRIL* has been found to be up-regulated in leukemia, prostate cancer, basal cell carcinoma and glioma, whereas depletion of *ANRIL* has been implicated in reduced proliferation, indicating its role in cancerogenesis [51-53]. Remarkably, in our study, eight major *ANRIL* isoforms, including the isoforms known to form circular variants (*cANRIL*), were found to be universally down-regulated in both iCD and iUC. Importantly, endogenous expression of *cANRIL* has been associated with risk for atherosclerosis [54]. In this context, dysregulation of *ANRIL* in IBD is highly intriguing, particularly the down-regulation of the *cANRIL* isoform. Indeed, recently, circular RNAs have been shown to be involved in stabilizing sense transcripts and also act as sponges for microRNAs [55]; however, the biological functions of circRNAs have recently been debated [56]. It is imperative, therefore, to investigate comprehensively the potential roles of *cANRIL* in IBD pathogenesis.

Unsurprisingly, our results also enabled us to distinguish between iCD and iUC, although the number of differentially expressed genes was small, which emphasizes the close pathogenic nature of CD and UC. An interesting distinction between iCD and iUC involved the expression of *SERPINB3* (serpin peptidase inhibitor, clade B (ovalbumin), member 3), which was significantly down-regulated (FC < -3.8) in iCD versus iUC (Table 6 and Figure 1G). *SERPINB3* has been found to be over-expressed in certain squamous epithelial cancers, such as uterine cervix carcinoma, head and neck carcinomas, and esophagus carcinoma [57]. Although the precise physiological functions of *SERPINB3* are elusive, it has been hypothesized that *SERPINB3* might be involved in the development of autoimmunity [58].

In our study, we found significant enrichment for the 96 differentially expressed lncRNAs within IBD loci. Collectively, we found differentially expressed IBD loci-associated lncRNAs overlapping active regulatory elements within known binding motifs in intestinal epithelium and immune cells [7]. LncRNA *RP3-395 M20.8* was found to be associated with the regulatory IBD risk variant rs10797432, which affects the binding motifs for *AP-2* (transcription factor AP-2 alpha (activating enhancer binding protein 2 alpha)) and *CTCF* (CCCTC-binding factor (zinc finger protein)) (Table S5 in Additional file 1). Moreover, IBD risk variant rs1569723 is known to act as a *cis*-eQTL for *CD40* (CD40 molecule, TNF receptor superfamily member 5), which was significantly up-regulated in iUC and associated with lncRNA *RP11-465 L10.10.* Additionally, lncRNA *IFNG-ASI* harboring UC susceptibility SNP rs7134599 was found to be up-regulated in iUC. SNP rs7134599 is associated with the IBD26 (12q15) genetic locus and with regulatory pro-inflammatory cytokines *IFNG* (interferon, gamma) and *IL-2* (interleukin 2) and anti-inflammatory cytokine *IL-26* (interleukin 26). *IFNG* gene encodes interferon gamma (*IFN-*γ), a soluble cytokine that is pivotal for the host’s innate and adaptive immunity against viral, certain bacterial and protozoal infections. Aberrant expression of *IFN-*γ has been linked with a number of autoimmune and inflammatory diseases, and mucosal expression of *IFN-*γ is known to play a vital role in the pathogenesis of IBD [59]. IL-2 is encoded by the *IL2* gene and is involved in immune responses to microbial infections and intestinal inflammation activation in IBD. Anti-inflammatory *IL-26* has been shown to be overexpressed in CD [60]. These findings suggest potential involvement of differentially expressed lncRNAs overlapping the active regulatory elements in IBD pathogenesis.

Interestingly, we also found positive (r^2^ ≥ 0.5) and extremely positive (r^2^ ≥ 0.9) correlations between the overlapping as well as *cis*-neighboring differentially expressed IBD loci-associated lncRNA-protein-coding gene pairs. A strong positive correlation was observed between lncRNA *AC051649.12* and protein-coding gene *LSP1* (lymphocyte-specific protein 1) associated with IBD risk variant rs907611. SNP rs907611 affects the binding affinity of transcriptional factors *YY1* and *NF-muE1* and thus alters gene expression. It is plausible that the differentially expressed IBD loci-associated lncRNAs intersecting protein-coding genes somehow contribute to the regulation of the latter [61]. Taken together, these data suggest lncRNAs have a role in regulating the expression of IBD loci-associated genes.

Additionally, we also noticed dysregulation of AMPs and inflammatory response genes such as pro-inflammatory chemokines and cytokines in various clinical subgroups. For example, the key antimicrobial response genes *REG3A* (Regenerating islet-derived 3 alpha), *DEFA5* (Defensin, alpha 5, Paneth cell-specific) and *DEFA6* (Defensin, alpha 6, Paneth cell-specific), were >30-fold up-regulated specially in iCD versus control. Consistent with our results, *REG3A*, *DEFA5* and *DEFA6* have been shown previously to be significantly up-regulated and linked to Paneth cell metaplasia in IBD [62,63]. Mutations in the cytoplasmic pathogen recognition receptor *NOD2* (nucleotide-binding oligomerization domain containing 2) gene have been associated with ileal CD and Paneth cell dysfunction [64] and, importantly, *NOD2* was found to be up-regulated in both iCD and iUC. Concordant with the findings by Arijs *et al*. [62], we also found two AMPs, *DEFB1* and *NPY*, significantly down-regulated in both iCD and iUC. *IL15* (interleukin 15) was found specifically up-regulated in iCD but not in iUC, which supports the notion that it contributes to acute intestinal inflammation in CD [65].

For all the differentially expressed lncRNAs and protein-coding genes, we evaluated biological functional processes through analysis of GO terms based on ‘guilt-by-association’ and WGCNA. Unsurprisingly, we found enrichment for immune response, pro-inflammatory cytokine activity, extracellular matrix organization, and ion membrane transport genes (Tables S9, S11, S12 and S13 in Additional file 1). Given the idiopathic nature of IBD, the overall up-regulation of pro-inflammatory immune response-related gene expression could be largely due to the infiltrating immune cells, rather than the underlying disease phenotype. Indeed, persistent inflammation in CD and UC is known to be elicited by the activation of innate and adaptive immune cells by foreign antigens, which in turn produce and release pro-inflammatory cytokines that give rise to the vicious circle of inflammation, thereby leading to chronic tissue injury and epithelial damage [66]. Nevertheless, differentially expressed genes identified in non-inflamed samples (niCD and niUC) versus control (Table S3 and S4 in Additional file 1) might be disease specific. In summary, our findings suggest that dysregulated lncRNAs could be involved in IBD pathogenesis. However, these findings warrant systematic experimental follow-up in cellular and murine models with additional validation in a larger cohort in order to elucidate the role and biomarker potential of these dysregulated lncRNAs in IBD.

## Conclusions

We show that lncRNA expression profiling can be effectively used to stratify iCD and iUC from healthy controls. Additionally, our data indicate the underlying potential of lncRNA transcriptional signatures associated with clinical parameters as biomarkers for IBD.

## Additional files

Additional file 1: Tables S1, S2, S3 and S4. The top differentially expressed lncRNAs and protein-coding genes for inflamed versus non-inflamed tissues in CD and UC. **Table S1.** Top 10 up/down-regulated lncRNAs and protein-coding genes in iCD versus niCD. **Table S2.** Top 10 up/down-regulated lncRNAs and protein-coding genes in iUC versus niUC. **Table S3.** Common differentially expressed genes found between iCD versus control and niCD versus control. **Table S4.** Common differentially expressed genes between iUC versus control and niUC versus control. **Tables S5, S6 and S7.** **Table S5.** List of oligonucleotides used for validating microarray data using real-time PCR analysis for selected 15 genes. **Table S6.** Validation of microarray results by real-time PCR analysis for eight differentially expressed genes in iCD versus control and iUC versus control. **Table S7.** Validation of microarray results by real-time PCR analysis for five differentially expressed genes in iCD versus iUC. **Table S8:** 96 differentially expressed lncRNAs found significantly enriched within IBD-loci. **Table S9:** Functional annotation of differentially expressed lncRNAs based on the nearest neighbor approach. **Tables S10, S11, S12 and S13.** Weighted correlation network analysis (WGCNA). **Table S10.** Gene significance. The co-expression network identified the gene significance for the different clinical parameters. **Table S11.** Brown module - immune and inflammatory response: 4,216 genes in the module, of which 1,748 have an Entrez ID (considered in GO analysis). **Table S12.** Green module - small molecule trans-membrane transport: 2,934 genes in the module, of which 1,210 have an Entrez ID (considered in GO analysis). **Table S13.** Red module - anionic and cationic transport: 2,486 genes in the module, of which 1,173 have an Entrez ID (considered in GO analysis). Additional file 2: Figure S1. Pearson correlations calculated for the technical replicates (six samples analyzed in duplicates on separate chips: 16_2, 18_3, 27_2, 28_3, 47_3 and 21_2). **Figure S2.** Scatterplot matrices describing the variation explained by the first four principal components for 90 biopsy samples. **Figure S3.** Unsupervised hierarchical clustering of the most dynamic probes (coefficient of variance >0.05) targeting lncRNAs (S3A) and protein-coding genes (S3B) across the samples in different clinical subgroups. **Figure S4.** Log2 ratio and -log10 adjusted *P*-values plotted and represented as volcano plots for the non-inflamed tissue comparisons iCD versus niCD (S4A) and iUC versus niUC (S4B). **Figure S5.** Expression map of the top 40 differentially expressed lncRNAs and protein-coding genes in iCD versus iUC based on unsupervised hierarchical clustering. **Figure S6.** Expression map of the total differentially expressed lncRNAs and protein-coding genes in iCD versus controls (S6A) and iUC versus controls (S6B) (patients in red, controls in blue) based on unsupervised hierarchical clustering. **Figures S7, S8 and S9.** **Figure S7.** Dendrogram of samples and heatmap of clinical parameters. Linear regression model and weighted correlation network analysis (WGCNA) were used to investigate the impact of clinical parameters on disease diagnosis. **Figure S8.** Receiver operating characteristic (ROC) curve analysis for age **(a)**, sex **(b)**, disease index **(c)**, smoking **(d)** classification using differentially expressed lncRNAs in all five comparisons. **Figure S9.** Overlap of differentially expressed genes identified by LIMMA and SVM. **Figures S10 and S11.** **Figure S10.** Co-expression network is built by hierarchical clustering and Dynamic Tree Cut. Modules are clusters of highly interconnected genes. The ‘brown’, ‘green’ and ‘red’ modules are enriched for differentially expressed genes between iUC/iCD and control. **Figure S11.** Module significance is determined as the average absolute gene significance measure for all genes in a given module.

## Abbreviations

AMP: antimicrobial peptide
CD: Crohn’s disease
eQTL: expression quantitative trait locus
FC: fold change
FDR: false discovery rate
GO: Gene Ontology
IBD: inflammatory bowel disease
iCD: inflamed Crohn’s disease
IFN: interferon
IL: interleukin
iUC: inflamed ulcerative colitis
lncRNA: long non-coding RNA
ncRNA: non-coding RNA
niCD: non-inflamed Crohn’s disease
niUC: non-inflamed ulcerative colitis
PCA: principal component analysis
PCR: polymerase chain reaction
qPCR: quantitative PCR
RFE: recursive feature elimination
SNP: single-nucleotide polymorphism
SVM: Support Vector Machines
UC: ulcerative colitis
WGCNA: weighted correlation network analysis
