# Supplementary material for: Transcriptomic landscape of lncRNAs in inflammatory bowel disease
Source: Genome Med. 2015 May 13;7(1):39. doi: 10.1186/s13073-015-0162-2 (PMC4437449; doi:10.1186/s13073-015-0162-2)
Supplement: Additional file 1: Tables S1, S2, S3 and S4. — The top differentially expressed lncRNAs and protein-coding genes for inflamed versus non-inflamed tissues in CD and UC. Table S1. Top 10 up/down-regulated lncRNAs and protein-coding genes in iCD versus niCD. Table S2. Top 10 up/down-regulated lncRNAs and protein-coding genes in iUC versus niUC. Table S3. Common differentially expressed genes found between iCD versus control and niCD versus control. Table S4. Common differentially expressed genes between iUC versus control and niUC versus control. Tables S5, S6 and S7. Table S5. List of oligonucleotides used for validating microarray data using real-time PCR analysis for selected 15 genes. Table S6. Validation of microarray results by real-time PCR analysis for eight differentially expressed genes in iCD versus control and iUC versus control. Table S7. Validation of microarray results by real-time PCR analysis for five differentially expressed genes in iCD versus iUC. Table S8: 96 differentially expressed lncRNAs found significantly enriched within IBD-loci. Table S9: Functional annotation of differentially expressed lncRNAs based on the nearest neighbor approach. Tables S10, S11, S12 and S13. Weighted correlation network analysis (WGCNA). Table S10. Gene significance. The co-expression network identified the gene significance for the different clinical parameters. Table S11. Brown module - immune and inflammatory response: 4,216 genes in the module, of which 1,748 have an Entrez ID (considered in GO analysis). Table S12. Green module - small molecule trans-membrane transport: 2,934 genes in the module, of which 1,210 have an Entrez ID (considered in GO analysis). Table S13. Red module - anionic and cationic transport: 2,486 genes in the module, of which 1,173 have an Entrez ID (considered in GO analysis). [file 13073_2015_162_MOESM1_ESM.pdf]

Table S1

Top ten up-regulated lncRNAs and protein-coding genes in iCD vs niCD

**LncRNAs (up-regulated)**			**Protein coding genes (up-regulated)**		
**Gene name**	**Transcript Name**	**FC**	**Gene Name**	**Transcript**	**FC**
RP11-731F5.2	ENST00000460164.1	3.01	CHI3L1	NM_001276	10.63
MMP12	ENST00000532855.1	2.87	DUOXA2	NM_207581	8.04
MMP12	ENST00000326227.5	2.79	CXCL1	NM_001511	5.54
LINC01272	ENST00000445003.1	2.77	SAA1	NM_000331	5.42
RP11-44K6.2	ENST00000520185.1	2.68	DMBT1	NM_007329	4.81
RP11-465L10.10	ENST00000419897.1	2.53	CXCL2	NM_002089	4.51
RP11-44K6.4	ENST00000522970.1	2.51	MMP7	NM_002423	4.44
SAA2-SAA4	ENST00000524555.1	2.51	S100A9	NM_002965	4.43
LINC01291	ENST00000435984.1	2.16	TNFRSF6B	NM_003823	4.39
LINC01291	ENST00000377469.1	1.93	IL1RN	NM_173843	4.13
**LncRNAs (down-regulated)**			**Protein coding genes (down-regulated)**		
DPP10-AS1	ENST00000432658.1	-3.23	PCK1	NM_002591	-4.23
ANRIL (CDKN2B-AS1)	ENST00000428597.1	-2.72	SLC5A11	NM_052944	-3.19
ANRIL (CDKN2B-AS1)	ENST00000422420.1	-2.71	AQP7P1	NR_002817	-3.03
ANRIL (CDKN2B-AS1)	ENST00000581051.1	-2.6	TMIGD1	NM_206832	-3.02
ANRIL (CDKN2B-AS1)	ENST00000582072.1	-2.57	SLC1A7	NM_006671	-2.95
ANRIL (CDKN2B-AS1)	ENST00000585267.1	-2.55	GUCA2B	NM_007102	-2.69
ANRIL (CDKN2B-AS1)	ENST00000577551.1	-2.51	MEP1A	NM_005588	-2.61
ANRIL (CDKN2B-AS1)	ENST00000421632.1	-2.5	NAALADL1	NM_005468	-2.58
ANRIL (CDKN2B-AS1)	ENST00000580576.1	-2.49	NPY	NM_000905	-2.55
RP11-680F8.1	ENST00000560994.1	-2.25	TNNC2	NM_003279	-2.45

Table S2

Top ten up-regulated lncRNAs and protein-coding genes in iUC vs niUC

**LncRNAs (up-regulated)**			**Protein coding genes (up-regulated)**		
**Gene name**	**Transcript Name**	**FC**	**Gene Name**	**Transcript**	**FC**
RP11-731F5.2	ENST00000460164.1	13.41	CHI3L1	NM_001276	29.95
MMP12	ENST00000532855.1	7.7	DUOXA2	NM_207581	29.39
MMP12	ENST00000326227.5	7.56	SAA1	NM_000331	21.99
RP11-465L10.10	ENST00000419897.1	6.41	CXCL1	NM_001511	14.8
SAA2-SAA4	ENST00000524555.1	5.36	MMP7	NM_002423	14.01
KIF9-AS1	ENST00000429315.2	4.28	SLC6A14	NM_007231	12.28
RP11-1149O23.3	ENST00000517774.1	4.17	IGHG3	ENST00000390551	11.59
FAM66D	ENST00000526690.1	4.06	TNFRSF6B	NM_003823	9.66
CLRN1-AS1	ENST00000476886.1	3.82	S100A9	NM_002965	8.83
LINC00484	ENST00000588550.1	3.68	OLFM4	NM_006418	8.81
**LncRNAs (down-regulated)**			**Protein coding genes (down-regulated)**		
ANRIL (CDKN2B-AS1)	ENST00000422420.1	-8.07	GUCA2A	NM_033553	-10.99
ANRIL (CDKN2B-AS1)	ENST00000428597.1	-7.98	TMIGD1	NM_206832	-10.84
ANRIL (CDKN2B-AS1)	ENST00000585267.1	-6.92	SLC26A2	NM_000112	-10.07
ANRIL (CDKN2B-AS1)	ENST00000580576.1	-6.79	PCK1	NM_002591	-9.85
ANRIL (CDKN2B-AS1)	ENST00000581051.1	-6.75	GUCA2B	NM_007102	-8.86
ANRIL (CDKN2B-AS1)	ENST00000577551.1	-6.63	ABCB1	NM_000927	-6.14
ANRIL (CDKN2B-AS1)	ENST00000582072.1	-6.54	UGT2B11	NM_001073	-5.62
ANRIL (CDKN2B-AS1)	ENST00000421632.1	-5.91	TRPM6	NM_017662	-5.32
DPP10-AS1	ENST00000432658.1	-5.2	OTOP2	NM_178160	-4.96
RP13-497K6.1	ENST00000506514.1	-4.63	PADI2	NM_007365	-4.95

Table S3

List of common up- and down-regulated lncRNAs and protein-coding genes between iCD vs control and niCD vs control

Gene name	Transcript Name	FC (niCD_vs_control)	FC (iCD_vs_control)
**LncRNAs (up-regulated)**			
ENSG00000253364	ENST00000460164.1	4.71	14.14
ENSG00000254802	ENST00000525556.1	1.52	2.53
ENSG00000204261	ENST00000413039.1	1.67	2.31
ENSG00000204261	ENST00000453426.1	1.54	2.03
ENSG00000204261	ENST00000415067.1	1.52	2.02
ENSG00000204261	ENST00000429600.1	1.53	1.97
ENSG00000259834	ENST00000566942.1	1.74	1.85
ENSG00000232788	ENST00000458314.1	1.51	1.54
**Protein-coding genes (up-regulated)**			
DUOXA2	NM_207581	5.88	47.26
ENST00000390551	ENST00000390551	3.83	11.52
IGFBP5	NM_000599	2.32	6.67
C4B	NM_001002029	2.32	5.46
ENST00000390625	ENST00000390625	2.6	4.77
ENST00000390603	ENST00000390603	2.6	4.67
ENST00000424969	ENST00000424969	2.58	4.41
ENST00000390600	ENST00000390600	2.43	4.04
C2	NM_000063	2.14	3.88
ENST00000491977	ENST00000491977	2.39	3.73
PIM2	NM_006875	1.91	3.67
IFITM4P	NR_001590	1.65	3.65
CD38	NM_001775	2.15	3.65
ENST00000390617	ENST00000390617	2.27	3.64
ENST00000354689	ENST00000354689	2.27	3.64
ENST00000390602	ENST00000390602	2.3	3.56
IFITM3	NM_021034	1.67	3.55
ENST00000390268	ENST00000390268	2.16	3.5
C1R	NM_001733	1.98	3.46
ENST00000492167	ENST00000492167	2.33	3.41
ENST00000390297	ENST00000390297	2.4	3.38
ENST00000390243	ENST00000390243	2.26	3.36
ENST00000390606	ENST00000390606	2.16	3.31
ENST00000453166	ENST00000453166	2.17	3.31
ENST00000390247	ENST00000390247	2.16	3.2
WNT5A	NM_003392	1.99	3.17
LOC100653210	ENST00000483158	2.18	3.15

S1PR4	NM_003775	1.99	3.08
ENST00000390294	ENST00000390294	2.22	3.04
SLAMF7	NM_021181	1.91	2.87
LUM	NM_002345	1.91	2.73
GBP3	NM_018284	2.62	2.71
CD74	NM_001025158	1.89	2.5
C1S	NM_201442	1.7	2.46
GIMAP4	NM_018326	1.83	2.46
GMFG	NM_004877	1.77	2.28
SLAMF1	NM_003037	1.64	2.23
IFFO1	NM_001039670	1.6	2.18
RGS18	NM_130782	1.73	2.16
ICAM2	NM_000873	1.56	2.13
GIMAP7	NM_153236	1.84	2.1
GIMAP5	NM_018384	1.65	2.08
IFNAR2	NM_000874	1.56	2.07
HLA-DPA1	NM_001242524	1.69	2.04
TRIB3	NM_021158	1.54	2
SUSD3	NM_145006	1.87	1.99
AMICA1	NM_153206	1.73	1.96
CECR1	NM_177405	1.64	1.95
BTN3A1	NM_007048	1.53	1.93
GIMAP6	NM_024711	1.64	1.89
VCAM1	NM_001078	1.65	1.88
CD40LG	NM_000074	1.87	1.83
CPVL	NM_019029	1.67	1.8
CCDC102A	NM_033212	1.51	1.72
TRAF3IP3	NM_025228	1.7	1.69
MFAP4	NM_002404	1.63	1.57
**LncRNAs (down-regulated)**			
ENST00000555403	ENST00000555403.1	-1.58	-2
ENST00000567599	ENST00000567599.1	-1.6	-1.98
ENST00000569655	ENST00000569655.1	-1.55	-1.7
ENST00000555282	ENST00000555282.1	-1.65	-1.52
**Protein-coding genes (down-regulated)**			
KCNK10	NM_021161	-2.01	-2.44
APOH	NM_000042	-1.84	-2.4
SSTR2	NM_001050	-2.03	-2.28
NT5E	NM_002526	-1.51	-1.74

Table S4

List of common up- and down-regulated lncRNAs and protein-coding genes between iUC vs control and niUC vs control

Gene name	Transcript Name	FC niUC vs control	FC iUC vs control
**LncRNAs (up-regulated)**			
ENST00000442197	ENST00000442197.1	2.17	1.72
**Protein-coding genes (up-regulated)**			
DPY19L1	NM_015283	1.57	2.04
IL6ST	NM_002184	1.54	1.84
**LncRNAs (down-regulated)**			
ENST00000563780	ENST00000563780.1	-4.48	-3.47
ENST00000571724	ENST00000571724.2	-2.02	-2.4
ENST00000592845	ENST00000592845.1	-1.61	-1.95
ENST00000590902	ENST00000590902.1	-1.6	-1.91
ENST00000592738	ENST00000592738.1	-1.57	-1.82
ENST00000580385	ENST00000580385.1	-1.83	-1.78
ENST00000586377	ENST00000586377.1	-1.53	-1.75
ENST00000542933	ENST00000542933.1	-1.74	-1.63
ENST00000432521	ENST00000432521.2	-1.78	-1.54
ENST00000448624	ENST00000448624.2	-1.83	-1.53
**Protein-coding genes (down-regulated)**			
MST1	NM_020998	-5.02	-3.77
CDK20	NM_001039803	-1.77	-2.86
RGS11	NM_003834	-2.27	-2.61
LPIN3	NM_022896	-1.9	-2.56
SPHK2	NM_020126	-1.76	-2.11
SFXN5	NM_144579	-1.53	-1.76
FAM73B	NM_032809	-1.51	-1.76
ACADVL	NM_000018	-1.71	-1.71
MICALL2	NM_182924	-1.89	-1.7
LOC338799	NR_002809	-1.78	-1.6
C6orf26	NM_001039651	-1.76	-1.59
LOC115110	NR_026927	-1.86	-1.56

Table S5

List of oligonucleotides used for validating microarray data using real–time PCR analysis for selected 15 genes

Gene name	Probe	Forward primer	Reverse primer
GAPDH	/56-FAM/AAG GTC GGA /ZEN/GTC AAC GGA TTT GGT C/3IABkFQ/	TGT AGT TGA GGT CAA TGA AGG G	ACA TCG CTC AGA CAC CAT G
KCNK10	/56-FAM/CTC ACA CAG /ZEN/ACA TGA TCC CGC AGG /3IABkFQ/	CGC ATT GTC AGC ATC AAG AG	GAG CAG CCA GAA GAA TAC CAT
DUOXA2	/56-FAM/CAA GCT TCC /ZEN/TGC TCA TCT TGC CG/3IABkFQ/	GTG CAC AGC CAC AAT TTC TG	CCA CTG CTC ATC GTT ATT CTA GT
DST	/56-FAM/TCT CCA GTG /ZEN/AAA CGC CGA AGA ATG C/3IABkFQ/	CTC TTG AAT AAT GAG ATC TGC TGA AC	GAA CAG TAC TCA GCT ACA GTG A
CHI3L1	/56-FAM/TGT CTG TCG /ZEN/GAG GAT GGA ACT TTG G/3IABkFQ/	TCT GGG TGT TGG AGG CTA T	GCT CAA CAC ACT CAA GAA CAG
DUOX2	/56-FAM/CCT GCA TAT /ZEN/TCC CCA ACG TCT TTG TG/3IABkFQ/	GAA GCA CAC CTG TCA TAC CTG	TTC TCA GTC AGC CCA CTC A
SERPINB3	/56-FAM/ACT TTG ATC /ZEN/AAG TCA CAG AGA ACA CCA CAG /3IABkFQ/	GTG ATG AAC ATT TCC TGA CCT	CAA AGA CAA CAC TGC ACA ACA G
MMP12	/56-FAM/TCT TTT GGG /ZEN/TCT CCA TAC AGG GAC TGA /3IABkFQ/	CAA AAC TCA AAT TGG GGT CAC AG	CTC TCT GCT GAT GAC ATA CGT G
PCK1	/56-FAM/CTG GGA ACC /ZEN/TGG CAT TGA ACG C/3IABkFQ/	CAT GCT GAA TGG GAT GAC GTA	GGA TGT CAG AGG AGG ATT TTG AG
RNA18S5	/56-FAM/TGC TCA ATC /ZEN/TCG GGT GGC TGA A/3IABkFQ/	GGA CAT CTA AGG GCA TCA CAG	GAG ACT CTG GCA TGC TAA CTA G
RP11-731F5.2	/56-FAM/AAG ACA AAT /ZEN/AGC AGC TGA CGG CGT /3IABkFQ/	TCT TTG CAG CGT GGA GTT	GTC TCA GCC CTT CCT GTT T
AC007182.6	/56-FAM/TGG CCT TTC /ZEN/CCA TGA GCT CTT CTG /3IABkFQ/	GCA TCA GAA CAA GCA GCA ATA A	CAA GTC CTG AGA CAG CTA GAA AT
AL928742.12	/56-FAM/TGG ACC GGC /ZEN/CTC TGC ATC TT/3IABkFQ/	CCA GAG CTG GAC GTG TG	CAC GCT GAG CAG GAA GAG
DPP10-AS1	/56-FAM/TGC TTC GGT /ZEN/TCC AAG TTT CAG GAA /3IABkFQ/	GGG AAG CAT GGG TCA GAA TAA	GAA GCT CCT GAA TCA CTG TAT GA
CDKN2B-AS1	/56-FAM/ATG AGA AAC /ZEN/AGA CAT GCT CCC TCC C/3IABkFQ/	GTC TCC ACA CTA AGC TGT AAC C	GAC TAC AGA TGC ACC ACC AT

Table S6

Validation of microarray results by Real-time PCR analysis for 8 differentially expressed genes in iCD vs control and iUC vs control. The up-regulated genes are highlighted in "red", and down-regulated genes in "green".

	iCD vs control		iUC vs control	
**Gene name**	**log2 FC**	**SD**	**log2 FC**	**SD**
**DUOXA2**	8.83	0.08	9.14	0.35
**CHI3L1**	9.79	0.22	8.51	0.40
**DUOX2**	5.85	0.12	6.05	0.35
**MMP12**	5.34	0.08	3.70	0.46
**RP11-731F5.2**	4.14	0.13	2.45	0.45
**PCK1**	-1.72	0.25	-2.33	0.34
**DPP10-AS1**	-2.97	0.27	-3.40	0.36
**ANRIL**	0.17	0.74	-0.31	0.33

Table S7

Validation of microarray results by Real-time PCR analysis for 5 differentially expressed genes in iCD vs iUC (*AC007182.6, AL928742.12, SERPINB3*), niCD vs control (*KCNK10*) and niUC vs control (*DST*). The up-regulated genes are highlighted in "red", and down-regulated genes in "green".

**Gene name**	**log2 FC**	**SD**
**iCD vs iUC**		
**AC007182.6**	2.46	0.05
**AL928742.12**	1.05	0.28
**SERPINB3**	-6.62	0.98
**niCD vs control**		
**KCNK10**	-0.16	0.30
**niUC vs control**		
**DST**	0.71	0.36

Table S8

96 differentially expressed lncRNAs found enriched within IBD-loci (500 kb long genomic regions with the IBD risk variant in the middle). For each IBD risk variant, the associated differentially expressed lncRNA is displayed along with its cis- neighboring protein-coding gene, cis-eQTL signals and affected motifs. The IBD risk variants co-localized with active regulatory elements in intestinal epithelium and immune cells are colored in red.

LncRNAs overlapping regulatory IBD loci	IBD loci (250 kb up/down marker SNP)		RegulomeDB Score	Affected binding motif	cis-eQTL (Affected gene)	Associated Protein coding genes	Differentially Expressed Protein coding genes
	chr	rsid					
ENST00000443892<br>ENST00000449660<br>ENST00000432521<br>ENST00000448624	chr1	rs10797432	1f	AP-2, CTCF	MELL1	MMEL1, PLCH2, TNFRSF14 , RP3-395M20.7	
ENST00000317726<br>ENST00000589757	chr19	rs11879191	1f		CDC37	CDC37, TYK2, PPAN-P2RY11, ICAM1	
ENST00000448685<br>ENST00000454321	chr10	rs12722515	7			IL2RA, IL15RA, LRG_73, RP11-536K7.5	IL15RA, IL2RA
ENST00000566140<br>ENST00000588180<br>ENST00000589987<br>ENST00000590346<br>ENST00000592556<br>ENST00000587298<br>ENST00000590012<br>ENST00000589777	chr17	rs1292053	6			TUBD1, NDUFB8P2, RPS6KB1	
ENST00000578478<br>ENST00000488188	chr17	rs12946510	1b	FOXO1, IRF1, Elf3, Srf	KRT222P, MED24,NR1D1,	IKZF3, ZPBP2, GSDMB, ORMDL3,	IKZF3, CSF3, THRA

					ORMDL3	GSDMA, RP11-94L15.2	
ENST00000442524	chr2	rs12994997	5			ATG16L1, INPP5D	DGKD
ENST00000558097<br>ENST00000560662	chr15	rs17293632	2a	AP-1,Bach1, Pou3f1, Sox5, Jundm2		SMAD3, RP11-342M21.2	
ENST00000520324<br>ENST00000523617	chr5	rs17695092	6			CPEB4	
ENST00000417795<br>ENST00000437091<br>ENST00000457998	chr5	rs2188962	2a	HNF4, LF-A1		IRF1, IL13, CSF2, SLC22A4, IL4, IL3, IL5, PDLIM4, SLC22A5, ACSL6, IBD5, C5orf56	SLC22A5
ENST00000449602	chr21	rs2823286	2b	p300			
ENST00000447729	chr17	rs3091316	7			CCL7, CCL2, CCL11, CCL8, CCL13, CCL1, CCL2	CCL11
ENST00000563780	chr3	rs3197999	7			PFKFB4, MST1R, UCN2, IP6K2, BSN, IP6K1, USP4, MST1, GPX1, APEH, AC099668.5	MST1, GMPPB, UBA7
ENST00000601909	chr1	rs4845604	4			RORC	
ENST00000357590<br>ENST00000538950<br>ENST00000545985<br>ENST00000330775<br>ENST00000526275<br>ENST00000532085<br>ENST00000527992<br>ENST00000456881<br>ENST00000530407<br>ENST00000524428<br>ENST00000525039<br>ENST00000525372	chr11	rs630923	1f		HINFP	RP11-158I9 . 5, CXCR5	
ENST00000454974	chr1	rs670523	7			KIAA0907, UBQLN4, RIT1, MSTO1	SYT11
ENST00000431144	chr6	rs6920220	6			OLIG3, TNFAIP3	
ENST00000541715	chr12	rs7134599	5			IFNG, IL26, IL22,	

ENST00000536914<br>ENST00000541707						GS1-410F4.2	
ENST00000584252	chr18	rs7240004	4			SMAD7	
ENST00000423967	chr21	rs7282490	7			ICOSLG	C21orf33
ENST00000566143	chr16	rs7404095	1f		PRKCB, PRKCB1	PRKCB	
ENST00000557339	chr14	rs8005161	5			GALC, GPR65, RP11-300J18.2	GPR65
ENST00000509204<br>ENST00000411861<br>ENST00000412788<br>ENST00000414790<br>ENST00000439725<br>ENST00000442037<br>ENST00000535745<br>ENST00000422826<br>ENST00000446406<br>ENST00000417089<br>ENST00000411754<br>ENST00000431095<br>ENST00000436715<br>ENST00000428066<br>ENST00000447298	chr11	rs907611	2a	YY1, NF-muE1		LSP1, TNNI2	LSP1
ENST00000445003	chr20	rs913678	4			CEBPB, COX6CP2	
ENST00000339009	chr13	rs9557195	4	Adf-1 , RREB1		UBAC2, GPR18, GPR183	GPR18
ENST00000417691<br>ENST00000422740	chr2	rs10181042	6			5S_rRNA, C2orf74, REL, PUS10	AHSA2
ENST00000599411	chr16	rs10521318	5			RP11-542M13.2, IRF8	
ENST00000413439<br>ENST00000594721	chr19	rs11672983	6			U6, NLRP7, NLRP2, LILRB4, KIR3DL2, FCAR, KIR3DL1	NLRP7
ENST00000417795<br>ENST00000437091<br>ENST00000457998	chr5	rs12521868	6			SLC22A4, SLC22A5, IRF1, IL3, C5orf56	SLC22A5
ENST00000317726<br>ENST00000589757	chr19	rs12720356	5			TYK2, ICAM1, ICAM3, LRG_121	
ENST00000448685<br>ENST00000454321	chr10	rs12722489	7			LRG_73, IL2RA	IL15RA, IL2RA

ENST00000449602	chr21	rs1297265	2b	AP-4, HEB, TAL1			
ENST00000567599<br>ENST00000352271	chr2	rs13428812	6			DNMT3A	POMC
ENST00000419897	chr20	rs1569723	1f		CD40	CD40, MMP9, PLTP, LRG_40	CD40
ENST00000599411	chr16	rs16940202	5			RP11-542M13 . 2	
ENST00000449602	chr21	rs1736020	5				
ENST00000593041	chr19	rs17694108	5			CEBPG	
ENST00000414046	chr6	rs1799964	4			LTA, HLA-DQA2, TNF, LST1, LTB	LTB, NCR3, APOM, DDAH2
ENST00000446887	chr6	rs212388	4			TAGAP, RP1-111C20 . 4, RP1-111C20.3	TAGAP
ENST00000435317	chr10	rs2227564	1f		ECD	PLAU, C10orf55	NDST2, PLAU
ENST00000423967	chr21	rs2838519	4			ICOSLG	
ENST00000488188<br>ENST00000578478	chr17	rs2872507	6			IKZF3, ORMDL3, IKZF3, PNMT, ZPBP2, GSDML	IKZF3, CSF3, THRA
ENST00000447729	chr17	rs3091315	5			CCL7, CCL2	CCL11
ENST00000520324<br>ENST00000523617	chr5	rs359457	6			CPEB4	
ENST00000442524	chr2	rs3792109	7			ATG16L1, SCARNA6	DGKD
ENST00000498745	chr7	rs4728142	1f		IRF5, TNPO3	KCP, IRF5, TNPO3, TSPAN33	TSPAN33
ENST00000588550	chr9	rs4743820	7			NFIL3, RP11-305L7 . 3, RP11-305L7.5, C9orf73, RP11-305L7.6	
ENST00000544553<br>ENST00000538355<br>ENST00000539086	chr11	rs559928	1f		FLJ37970	CCDC88B, RPS6KA4, TRPT1, FLRT1	FERMT3, ESRRA, CCDC88B
ENST00000419244	chr2	rs6740462	4			AC074391 . 1, SPRED2, AC012370.3	
ENST00000443574<br>ENST00000453426<br>ENST00000458296<br>ENST00000413039<br>ENST00000429600	chr6	rs6927022	6			HLA-DQB1, HLA-DQA1, HLA-DRA, HLA-DQA2, HLA-DRB1	HLA-DQA1, HLA-DQB1, HLA-DQA2, HLA-DQB2, TAP2

ENST00000412095<br>ENST00000415067							
ENST00000544553<br>ENST00000538355<br>ENST00000539086	chr11	rs694739	1f	TBX5	CCDC88, FLJ37970	ESRRA, AP003774 . 1, PRDX5	FERMT3, ESRRA, CCDC88B
ENST00000457958<br>ENST00000577828<br>ENST00000580948	chr17	rs7210086	7			SLC39A11, CTD-3010D24 . 3	
ENST00000443892<br>ENST00000449660<br>ENST00000448624<br>ENST00000432521	chr1	rs734999	1f		TNFRSF14	RP3-395M20 . 9, TNFRSF14, MMEL1, PLCH2, C1orf93, FAM213B	
ENST00000593041	chr19	rs736289	5				
ENST00000417691<br>ENST00000422740	chr2	rs7608910	6			REL, C2orf74, KIAA1841, AHSA2, PUS10	AHSA2
ENST00000414046	chr6	rs9264942	6			XXbac-BPG248L24 . 10, HLA-C,PSORS1C1, NFKBIL1,MICB, HLA-C, XXbac-BPG248L24.13	LTB, NCR3, APOM, DDAH2
ENST00000443574	chr6	rs9268853	6			HLA-DRB9, HLA-DRB5, HLA-DQA1, HLA-DRB1, HLA-DRA, BTNL2, MHC, BTLN2	HLA-DQA1, HLA-DQB1
ENST00000563780	chr3	rs9822268	5			MST1, APEH, AC099668.5, UBA7, AMIGO3, GMPPB, BSN	MST1, GMPPB, UBA7

Table S9

Functional Annotation of differentially expressed lncRNAs based on nearest neighbor approach. The table lists over-represented GO terms of genes that overlap with or are neighbors of the differentially expressed lncRNAs and IBD loci associated lncRNAs. The overrepresentation test is performed by comparing the input gene list to the reference list using the binomial test for each molecular function, biological process, or pathway term in PANTHER. Bonferroni correction for multiple testing was used.

Overrepresented GO term	Number of genes in the reference list	Number of genes mapped to the GO term	Expected	P value
**Biological Process**				
antigen processing and presentation	81	22	4.08	7.39E-08
antigen processing and presentation of peptide or polysaccharide antigen via MHC class II	51	17	2.57	3.65E-07
immune system process	1733	137	87.19	2.50E-05
response to stimulus	1671	132	84.07	4.53E-05
natural killer cell activation	103	20	5.18	9.46E-05
cellular defense response	387	44	19.47	1.64E-04
immune response	632	60	31.8	6.40E-04
cell communication	3221	215	162.05	1.51E-03
cellular process	5952	354	299.46	2.73E-02
cell-cell signaling	835	66	42.01	4.96E-02
**Molecular Function**				
protein binding	2855	197	143.64	4.70E-04
receptor binding	1017	79	51.17	1.99E-02
cytokine activity	188	22	9.46	5.01E-02
**Cellular Component**				
MHC protein complex	42	17	2.11	5.95E-09
**PANTHER pathway**				
T cell activation	82	14	4.13	1.78E-02

Table S10

Gene significance: The co-expression network identified the gene significance for the different clinical traits. Genes are counted if gene significance p-value < 0.05. Differentially expressed genes between iUC / iCD and Control are found with LIMMA.

Clinical trait	All genes	Differentially expressed genes
Sex	5427	488
Age	7315	1006
Biopsy location	8029	585
Clinical subgroup	5459	307
Ethnicity	8921	949
Smoking	3655	204
Disease index	10435	509

Table S11

Brown module - Immune and inflammatory response: 4216 genes in the module of which 1748 have an Entrez ID (considered in GO analysis). 1300 genes are differentially expressed between iCD / iUC and control (880 iCD vs control; 1179 iUC vs control).

Count	GO ID	Term	p-value	adjusted p-value	Significant Genes	Total Genes
1	GO:0006955	immune response	5.2738E-028	3.291E-024	245	1323
2	GO:0002376	immune system process	9.7382E-028	3.291E-024	343	2128
3	GO:0009611	response to wounding	3.0972E-025	6.978E-022	217	1161
4	GO:0006952	defense response	2.625E-023	4.4355E-020	244	1414
5	GO:0006954	inflammatory response	5.0644E-022	6.8461E-019	124	541
6	GO:0001775	cell activation	3.729E-019	3.9381E-016	158	830
7	GO:0050896	response to stimulus	4.0785E-019	3.9381E-016	810	7012
8	GO:0048583	regulation of response to stimulus	6.3975E-019	5.405E-016	393	2839
9	GO:0002682	regulation of immune system process	4.095E-018	3.0754E-015	191	1108
10	GO:0045321	leukocyte activation	1.1585E-017	7.3941E-015	125	614
11	GO:0006950	response to stress	1.2034E-017	7.3941E-015	429	3224
12	GO:0007165	signal transduction	2.3438E-017	1.3201E-014	562	4529
13	GO:0022610	biological adhesion	2.8207E-017	1.4666E-014	176	1008
14	GO:0007155	cell adhesion	4.1947E-017	2.0252E-014	175	1004
15	GO:0023052	signaling	1.2779E-016	5.3981E-014	606	5006
16	GO:0044700	single organism signaling	1.2779E-016	5.3981E-014	606	5006
17	GO:0007154	cell communication	6.3527E-016	2.5258E-013	610	5081
18	GO:0009605	response to external stimulus	3.037E-015	1.1404E-012	269	1847
19	GO:0048584	positive regulation of response to stimulus	9.9456E-015	3.538E-012	219	1429
20	GO:0001816	cytokine production	2.97E-014	1.0037E-011	103	514
21	GO:0002684	positive regulation of immune system process	1.9178E-013	6.1727E-011	123	679
22	GO:0050776	regulation of immune response	8.0313E-013	2.4674E-010	132	763
23	GO:0046649	lymphocyte activation	1.1455E-012	3.3664E-010	100	521
24	GO:0002252	immune effector process	1.6345E-012	4.6031E-010	108	584
25	GO:0050865	regulation of cell activation	1.7513E-012	4.7349E-010	82	394
26	GO:0051716	cellular response to stimulus	3.264E-012	8.4851E-010	632	5509
27	GO:0002694	regulation of leukocyte activation	3.6127E-012	9.0438E-010	77	364
28	GO:0031347	regulation of defense response	4.1305E-012	0.000000001	96	502
29	GO:0007166	cell surface receptor signaling pathway	6.1087E-012	1.4238E-009	339	2617
30	GO:0048518	positive regulation of biological process	9.3846E-012	2.1144E-009	475	3944

31	GO:0030198	extracellular matrix organization	2.2982E-011	0.000000005	75	363
32	GO:0065007	biological regulation	2.3686E-011	0.000000005	968	9193
33	GO:0043062	extracellular structure organization	2.6391E-011	5.4054E-009	75	364
34	GO:0008283	cell proliferation	2.9314E-011	5.8274E-009	235	1687
35	GO:0045087	innate immune response	3.4811E-011	6.7226E-009	135	828
36	GO:0006935	chemotaxis	6.1037E-011	1.115E-008	109	626
37	GO:0042330	taxis	6.1037E-011	1.115E-008	109	626
38	GO:0070887	cellular response to chemical stimulus	1.1805E-010	0.000000021	272	2049
39	GO:0051239	regulation of multicellular organismal process	1.4578E-010	2.5265E-008	263	1970
40	GO:0040011	locomotion	1.743E-010	2.905E-008	196	1369
41	GO:0044763	single-organism cellular process	1.7622E-010	2.905E-008	1099	10756
42	GO:0009607	response to biotic stimulus	2.1861E-010	3.3822E-008	113	671
43	GO:0042110	T cell activation	2.1957E-010	3.3822E-008	75	380
44	GO:0001944	vasculature development	2.2018E-010	3.3822E-008	98	553
45	GO:0071310	cellular response to organic substance	2.5754E-010	3.8683E-008	224	1625
46	GO:0080134	regulation of response to stress	3.3784E-010	4.9641E-008	137	872
47	GO:0001817	regulation of cytokine production	4.4126E-010	6.3456E-008	85	460
48	GO:0042127	regulation of cell proliferation	4.752E-010	6.6913E-008	185	1288
49	GO:0042221	response to chemical	5.031E-010	6.9396E-008	382	3125
50	GO:0050727	regulation of inflammatory response	5.4047E-010	7.3061E-008	52	226
51	GO:0043207	response to external biotic stimulus	5.981E-010	7.9266E-008	108	642
52	GO:0050778	positive regulation of immune response	7.2221E-010	9.3126E-008	89	495
53	GO:0010033	response to organic substance	7.3024E-010	9.3126E-008	285	2206
54	GO:0048522	positive regulation of cellular process	1.0624E-009	0.000000133	419	3506
55	GO:0051707	response to other organism	1.3581E-009	1.669E-007	106	635
56	GO:0034097	response to cytokine	1.5886E-009	1.9174E-007	98	573
57	GO:0032101	regulation of response to external stimulus	2.2287E-009	2.6428E-007	94	545
58	GO:0044699	single-organism process	2.4102E-009	2.8087E-007	1198	12011
59	GO:0002274	myeloid leukocyte activation	2.5703E-009	2.9445E-007	36	133
60	GO:0051249	regulation of lymphocyte activation	2.7118E-009	3.0548E-007	64	320
61	GO:0001568	blood vessel development	2.8907E-009	3.2029E-007	78	424
62	GO:0019221	cytokine-mediated signaling pathway	3.5743E-009	3.8966E-007	70	366

63	GO:0050789	regulation of biological process	3.6325E-009	3.8972E-007	909	8685
64	GO:0071345	cellular response to cytokine stimulus	6.7034E-009	7.0794E-007	84	478
65	GO:0042060	wound healing	7.2995E-009	0.000000759	104	638
66	GO:0009966	regulation of signal transduction	8.2567E-009	8.4556E-007	274	2154
67	GO:0044707	single-multicellular organism process	8.3905E-009	8.4644E-007	619	5597
68	GO:0030155	regulation of cell adhesion	9.6911E-009	9.6327E-007	60	301
69	GO:0050794	regulation of cellular process	1.6081E-008	1.5752E-006	862	8216
70	GO:0048514	blood vessel morphogenesis	0.000000017	1.6387E-006	67	357
71	GO:0034341	response to interferon-gamma	1.8575E-008	1.7683E-006	32	118
72	GO:0050867	positive regulation of cell activation	3.2748E-008	3.0742E-006	52	253
73	GO:0072358	cardiovascular system development	3.9436E-008	0.000003602	112	725
74	GO:0072359	circulatory system development	3.9436E-008	0.000003602	112	725
75	GO:0010646	regulation of cell communication	4.9291E-008	4.4421E-006	297	2414
76	GO:0048870	cell motility	5.1869E-008	4.6129E-006	148	1038
77	GO:0023051	regulation of signaling	5.4838E-008	4.8136E-006	296	2407
78	GO:0006928	cellular component movement	7.5594E-008	6.4986E-006	201	1521
79	GO:0050863	regulation of T cell activation	0.000000076	6.4986E-006	50	245
80	GO:0016477	cell migration	8.2529E-008	6.9726E-006	138	958
81	GO:0001525	angiogenesis	9.1947E-008	7.6725E-006	68	380
82	GO:0007596	blood coagulation	1.0344E-007	8.5262E-006	84	507
83	GO:0050817	coagulation	1.3462E-007	0.000010963	84	510
84	GO:0001819	positive regulation of cytokine production	1.3936E-007	0.000011213	52	264
85	GO:0002696	positive regulation of leukocyte activation	1.5081E-007	0.000011992	49	243
86	GO:0050900	leukocyte migration	1.5879E-007	0.000012443	55	287
87	GO:0007599	hemostasis	1.6016E-007	0.000012443	84	512
88	GO:0009967	positive regulation of signal transduction	1.6852E-007	0.000012943	140	987
89	GO:0002460	adaptive immune response based on somatic recombination of immune receptors built from immunoglobulin superfamily domains	1.8107E-007	0.000013752	45	216
90	GO:0002443	leukocyte mediated immunity	0.000000191	0.000014341	50	252
91	GO:0050870	positive regulation of T cell activation	2.5893E-007	0.000019232	39	177
92	GO:0051674	localization of cell	2.6652E-007	0.00001958	107	709

93	GO:0010941	regulation of cell death	2.7922E-007	0.000020293	173	1291
94	GO:0043067	regulation of programmed cell death	2.8385E-007	0.00002041	168	1246
95	GO:0070661	leukocyte proliferation	3.1012E-007	0.00002174	46	227
96	GO:0050663	cytokine secretion	3.1059E-007	0.00002174	29	113
97	GO:0032501	multicellular organismal process	0.000000312	0.00002174	628	5812
98	GO:0065008	regulation of biological quality	3.7782E-007	0.00002597	336	2846
99	GO:0008219	cell death	3.8039E-007	0.00002597	234	1863
100	GO:0016265	death	4.1448E-007	0.000027971	234	1865
101	GO:0060326	cell chemotaxis	0.000000418	0.000027971	40	187
102	GO:0046651	lymphocyte proliferation	0.000000479	0.000031737	44	216
103	GO:0035556	intracellular signal transduction	5.1226E-007	0.000033615	256	2079
104	GO:0012501	programmed cell death	0.000000548	0.000035612	213	1674
105	GO:0071706	tumor necrosis factor superfamily cytokine production	5.9018E-007	0.000037991	23	80
106	GO:0042981	regulation of apoptotic process	5.9586E-007	0.000037995	165	1233
107	GO:0051251	positive regulation of lymphocyte activation	6.1229E-007	0.000038677	45	225
108	GO:0032943	mononuclear cell proliferation	6.2579E-007	0.000038885	44	218
109	GO:0071346	cellular response to interferon-gamma	6.2708E-007	0.000038885	26	98
110	GO:0008284	positive regulation of cell proliferation	0.000000633	0.000038894	108	730
111	GO:0002253	activation of immune response	6.7281E-007	0.000040969	70	416
112	GO:0060333	interferon-gamma-mediated signaling pathway	7.0678E-007	0.000042653	22	75
113	GO:0032609	interferon-gamma production	7.7787E-007	0.000046528	24	87
114	GO:0023056	positive regulation of signaling	9.7544E-007	0.000057833	142	1035
115	GO:0002263	cell activation involved in immune response	1.1065E-006	0.000064474	36	166
116	GO:0002366	leukocyte activation involved in immune response	1.1065E-006	0.000064474	36	166
117	GO:0010647	positive regulation of cell communication	1.2154E-006	0.000070211	142	1039
118	GO:0006915	apoptotic process	1.2354E-006	0.000070766	209	1655
119	GO:0048519	negative regulation of biological process	1.6868E-006	0.000095809	393	3457
120	GO:0042113	B cell activation	1.7088E-006	0.00009625	40	197
121	GO:0051240	positive regulation of multicellular organismal process	1.8929E-006	0.00010574	88	576
122	GO:0030334	regulation of cell migration	0.000002179	0.00012072	75	470

123	GO:0002824	positive regulation of adaptive immune response based on somatic recombination of immune receptors built from immunoglobulin superfamily domains	2.2113E-006	0.00012151	18	57
124	GO:0030574	collagen catabolic process	2.2827E-006	0.00012443	21	74
125	GO:0072507	divalent inorganic cation homeostasis	2.3158E-006	0.00012522	56	319
126	GO:0002521	leukocyte differentiation	2.3508E-006	0.0001261	65	390
127	GO:0007159	leukocyte cell-cell adhesion	2.7368E-006	0.00014566	15	42
128	GO:0044243	multicellular organismal catabolic process	0.000002951	0.00015583	22	81
129	GO:0002822	regulation of adaptive immune response based on somatic recombination of immune receptors built from immunoglobulin superfamily domains	3.3424E-006	0.00017513	25	100
130	GO:0032640	tumor necrosis factor production	3.6544E-006	0.00018855	21	76
131	GO:0032680	regulation of tumor necrosis factor production	3.6544E-006	0.00018855	21	76
132	GO:0002437	inflammatory response to antigenic stimulus	3.8447E-006	0.00019687	14	38
133	GO:0002449	lymphocyte mediated immunity	4.2395E-006	0.00021545	40	204
134	GO:0098542	defense response to other organism	4.3545E-006	0.00021899	60	357
135	GO:0009306	protein secretion	4.3739E-006	0.00021899	39	197
136	GO:0030098	lymphocyte differentiation	4.4975E-006	0.00022352	48	264
137	GO:0022617	extracellular matrix disassembly	4.5353E-006	0.00022375	28	121
138	GO:0031349	positive regulation of defense response	4.6876E-006	0.00022959	49	272
139	GO:0002697	regulation of immune effector process	4.7895E-006	0.0002329	47	257
140	GO:0002821	positive regulation of adaptive immune response	0.00000503	0.00024284	18	60
141	GO:0009615	response to virus	5.3924E-006	0.00025849	50	281
142	GO:0032732	positive regulation of interleukin-1 production	5.6351E-006	0.00026822	11	25
143	GO:0002250	adaptive immune response	5.6827E-006	0.0002686	46	251
144	GO:0009653	anatomical structure morphogenesis	5.9803E-006	0.0002807	261	2192
145	GO:0002237	response to molecule of	6.3922E-006	0.00029796	44	237

		bacterial origin				
146	GO:0002683	negative regulation of immune system process	6.9403E-006	0.0003213	40	208
147	GO:0032652	regulation of interleukin-1 production	7.7232E-006	0.00035511	14	40
148	GO:0032103	positive regulation of response to external stimulus	7.9394E-006	0.00036258	36	180
149	GO:0042098	T cell proliferation	8.2174E-006	0.00037276	31	145
150	GO:2000026	regulation of multicellular organismal development	8.4612E-006	0.00038126	161	1251
151	GO:2000145	regulation of cell motility	8.5642E-006	0.00038335	76	496
152	GO:1902531	regulation of intracellular signal transduction	8.8267E-006	0.0003925	165	1289
153	GO:0032496	response to lipopolysaccharide	8.9571E-006	0.00039569	42	225
154	GO:0032963	collagen metabolic process	9.2478E-006	0.00040588	26	112
155	GO:0030097	hemopoiesis	0.00001052	0.00045872	67	424
156	GO:0032731	positive regulation of interleukin-1 beta production	0.000010642	0.0004611	10	22
157	GO:0072091	regulation of stem cell proliferation	0.000010899	0.00046923	21	81
158	GO:0032651	regulation of interleukin-1 beta production	0.000011015	0.0004712	13	36
159	GO:0055074	calcium ion homeostasis	0.000011271	0.00047569	52	304
160	GO:0072503	cellular divalent inorganic cation homeostasis	0.000011271	0.00047569	52	304
161	GO:0050716	positive regulation of interleukin-1 secretion	0.000011331	0.00047569	9	18
162	GO:0050707	regulation of cytokine secretion	0.00001196	0.00049901	23	94
163	GO:0048534	hematopoietic or lymphoid organ development	0.000012803	0.0005309	72	468
164	GO:0030335	positive regulation of cell migration	0.000013694	0.00056436	47	267
165	GO:0016337	cell-cell adhesion	0.000013778	0.0005644	69	444
166	GO:0050866	negative regulation of cell activation	0.000014127	0.00057521	28	128
167	GO:0050793	regulation of developmental process	0.000014282	0.00057803	203	1658
168	GO:0007275	multicellular organismal development	0.000014475	0.00058236	466	4274
169	GO:0002819	regulation of adaptive immune response	0.000017052	0.00068199	25	109
170	GO:0031295	T cell costimulation	0.000017375	0.00069079	19	71
171	GO:0002703	regulation of leukocyte mediated immunity	0.000017998	0.00071139	26	116

172	GO:0050864	regulation of B cell activation	0.000018693	0.00073459	22	90
173	GO:0050715	positive regulation of cytokine secretion	0.000021197	0.00082817	17	60
174	GO:0031294	lymphocyte costimulation	0.00002157	0.0008353	19	72
175	GO:0010043	response to zinc ion	0.000021627	0.0008353	13	38
176	GO:0051336	regulation of hydrolase activity	0.000021922	0.00083762	124	932
177	GO:0044767	single-organism developmental process	0.000022039	0.00083762	520	4848
178	GO:0051607	defense response to virus	0.000022059	0.00083762	38	203
179	GO:0032623	interleukin-2 production	0.0000234	0.00088357	15	49
180	GO:0002695	negative regulation of leukocyte activation	0.000024737	0.00091912	26	118
181	GO:0044259	multicellular organismal macromolecule metabolic process	0.000024737	0.00091912	26	118
182	GO:2000147	positive regulation of cell motility	0.000024749	0.00091912	47	273
183	GO:0048731	system development	0.00002509	0.0009267	391	3530
184	GO:0002275	myeloid cell activation involved in immune response	0.000025676	0.00094317	16	55
185	GO:0032879	regulation of localization	0.000026558	0.00097031	195	1598
186	GO:0002706	regulation of lymphocyte mediated immunity	0.000027033	0.00098234	22	92

Table S12

Green module - Small molecule trans-membrane transport: 2934 genes in the module of which 1210 have an Entrez ID (considered in GO analysis). 397 genes are differentially expressed between iCD / iUC and control (132 iCD vs control; 311 iUC vs control).

Count	GO ID	Term	p-value	adjusted p-value	Significant Genes	Total Genes
1	GO:0044281	small molecule metabolic process	3.1826E-012	1.85E-008	268	2824
2	GO:0006629	lipid metabolic process	1.9167E-011	5.571E-008	137	1201
3	GO:0044710	single-organism metabolic process	9.1128E-009	0.000017658	390	4775
4	GO:0044699	single-organism process	2.5462E-008	0.000037003	841	12011
5	GO:0008202	steroid metabolic process	8.2418E-008	0.000081653	43	278
6	GO:0044763	single-organism cellular process	8.428E-008	0.000081653	766	10756
7	GO:0006082	organic acid metabolic process	1.3037E-007	0.00010827	110	1038
8	GO:0055085	transmembrane transport	0.00000018	0.0001308	99	911
9	GO:0006820	anion transport	2.4109E-007	0.00014038	54	402
10	GO:0046942	carboxylic acid transport	2.415E-007	0.00014038	37	230
11	GO:0015849	organic acid transport	3.0181E-007	0.00014708	37	232
12	GO:0019752	carboxylic acid metabolic process	3.0363E-007	0.00014708	98	909
13	GO:0006811	ion transport	4.6444E-007	0.00020767	120	1189
14	GO:0044255	cellular lipid metabolic process	5.9838E-007	0.00023864	95	886
15	GO:0043436	oxoacid metabolic process	6.1579E-007	0.00023864	106	1021
16	GO:0044765	single-organism transport	1.9629E-006	0.00071316	258	3085
17	GO:0015711	organic anion transport	2.1563E-006	0.00073731	44	323

Table S13

Red module - Anionic and cationic transport: 2486 genes in the module of which 1173 have an Entrez ID (considered in GO analysis). 357 genes are differentially expressed between iCD / iUC and control (185 iCD vs control; 311 iUC vs control).

Count	GO ID	Term	p-value	adjusted p-value	Significant Genes	Total Genes
1	GO:0022904	respiratory electron transport chain	4.2697E-019	2.3705E-015	36	100
2	GO:0022900	electron transport chain	9.1149E-019	2.5303E-015	36	102
3	GO:0045333	cellular respiration	5.7978E-017	1.073E-013	42	154
4	GO:0015980	energy derivation by oxidation of organic compounds	3.2818E-013	4.5551E-010	57	325
5	GO:1902600	hydrogen ion transmembrane transport	1.1287E-010	1.2533E-007	21	65
6	GO:0055114	oxidation-reduction process	4.3185E-010	3.996E-007	73	559
7	GO:0015992	proton transport	3.3957E-009	2.6933E-006	23	91
8	GO:0006091	generation of precursor metabolites and energy	4.6912E-009	3.2557E-006	57	412
9	GO:0006818	hydrogen transport	5.3516E-009	3.3013E-006	23	93
10	GO:0044281	small molecule metabolic process	1.9139E-008	0.000010626	238	2824
11	GO:0006811	ion transport	1.029E-007	0.000051938	117	1189
12	GO:0042773	ATP synthesis coupled electron transport	1.5874E-007	0.000065269	15	50
13	GO:0042775	mitochondrial ATP synthesis coupled electron transport	1.5874E-007	0.000065269	15	50
14	GO:0006810	transport	1.6458E-007	0.000065269	293	3713
15	GO:0051234	establishment of localization	2.0071E-007	0.000074291	297	3781
16	GO:0006820	anion transport	4.9169E-007	0.00017062	51	402
17	GO:0015985	energy coupled proton transport, down electrochemical gradient	0.00000059	0.00017757	9	19
18	GO:0015986	ATP synthesis coupled proton transport	0.00000059	0.00017757	9	19
19	GO:0034220	ion transmembrane transport	6.0769E-007	0.00017757	63	544
20	GO:0044765	single-organism transport	8.1734E-007	0.00022689	247	3085
21	GO:0055085	transmembrane transport	1.6494E-006	0.00043606	91	911
22	GO:0006754	ATP biosynthetic process	0.000002057	0.00051911	12	39
23	GO:0015672	monovalent inorganic cation transport	2.1905E-006	0.00052876	45	354
