# Supplementary material for: Transcriptomic landscape of lncRNAs in inflammatory bowel disease
Source: Genome Med. 2015 May 13;7(1):39. doi: 10.1186/s13073-015-0162-2 (PMC4437449; doi:10.1186/s13073-015-0162-2)

Figure S1

The Pearson correlations calculated for the technical replicated (six samples analyzed in duplicates on separate chips 16_2, 18_3, 27_2, 28_3, 47_3 and 21_2). For each technical replicate, the biopsy location and sex is also listed. Overall positive correlations ($r^2 \geq 0.9$) were observed between all technical replicates.

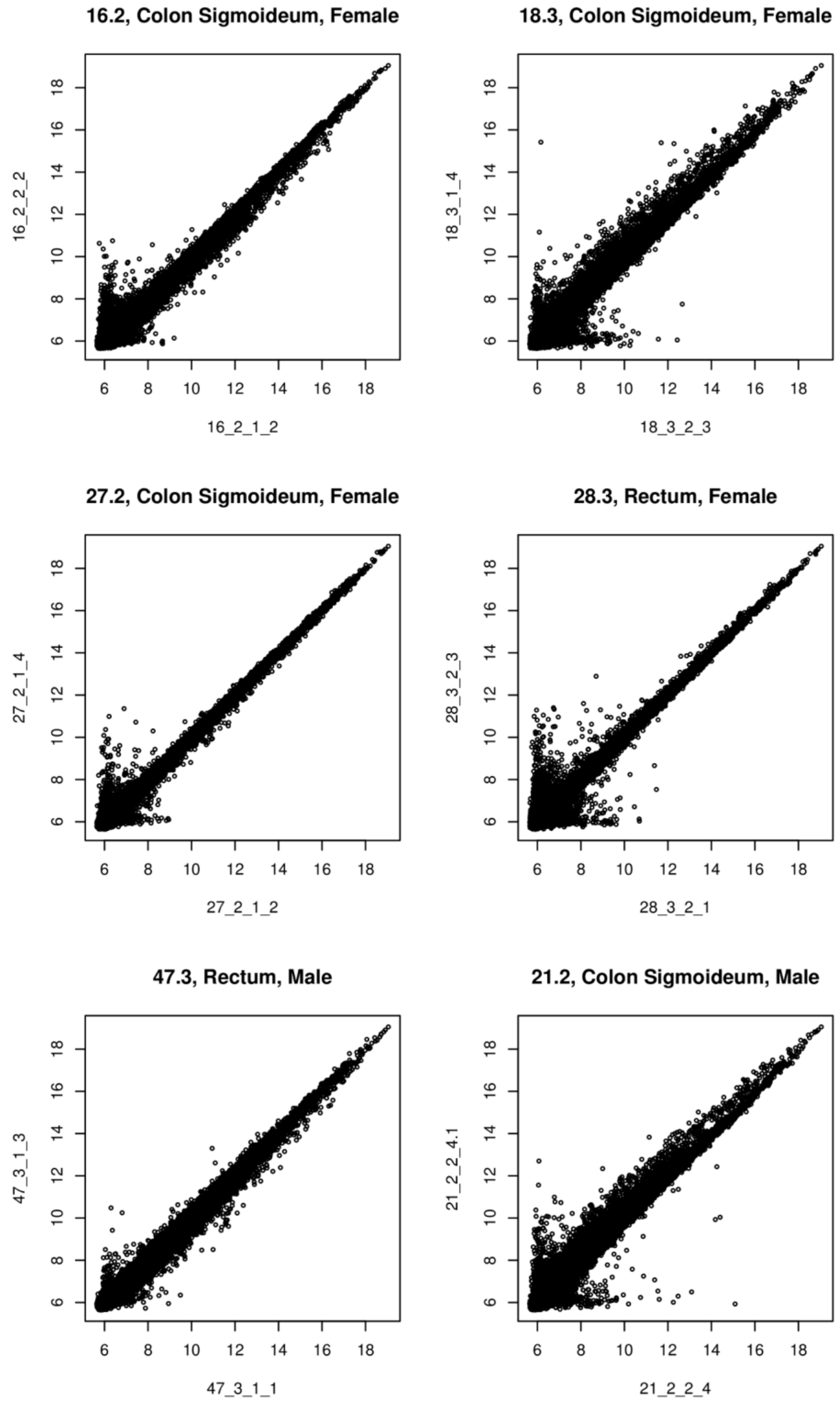

Figure S2

The scatterplot matrices describing the variation explained by the first four principal components for 90 biopsy samples. The different clinical subgroups iCD, iUC, niCD, niUC and controls are depicted in red, green, blue, cyan and black respectively.

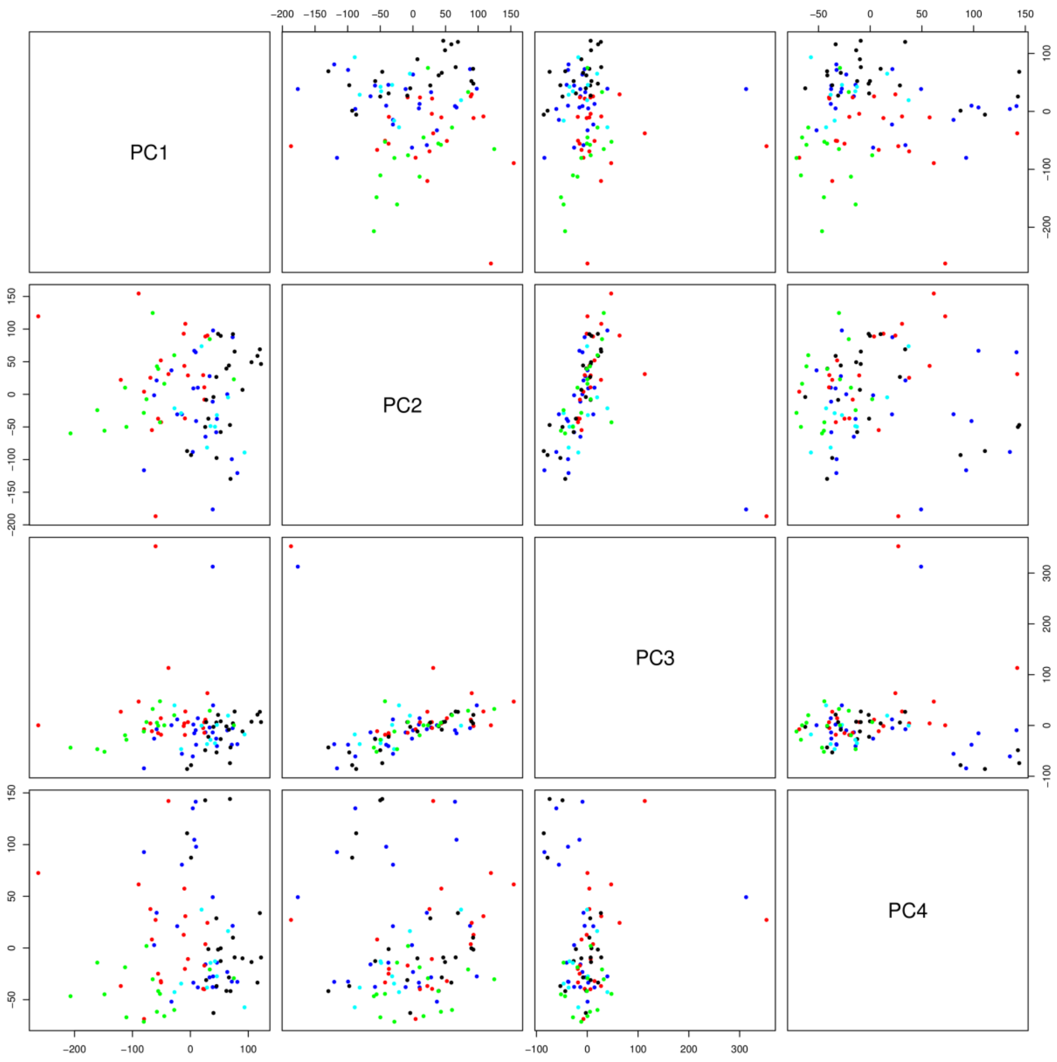

Figure S3

Unsupervised hierarchical clustering of the most dynamic probes (coefficient of variance >0.05) targeting lncRNAs (S3A) and protein-coding genes (S3B) across the samples in different clinical subgroups.

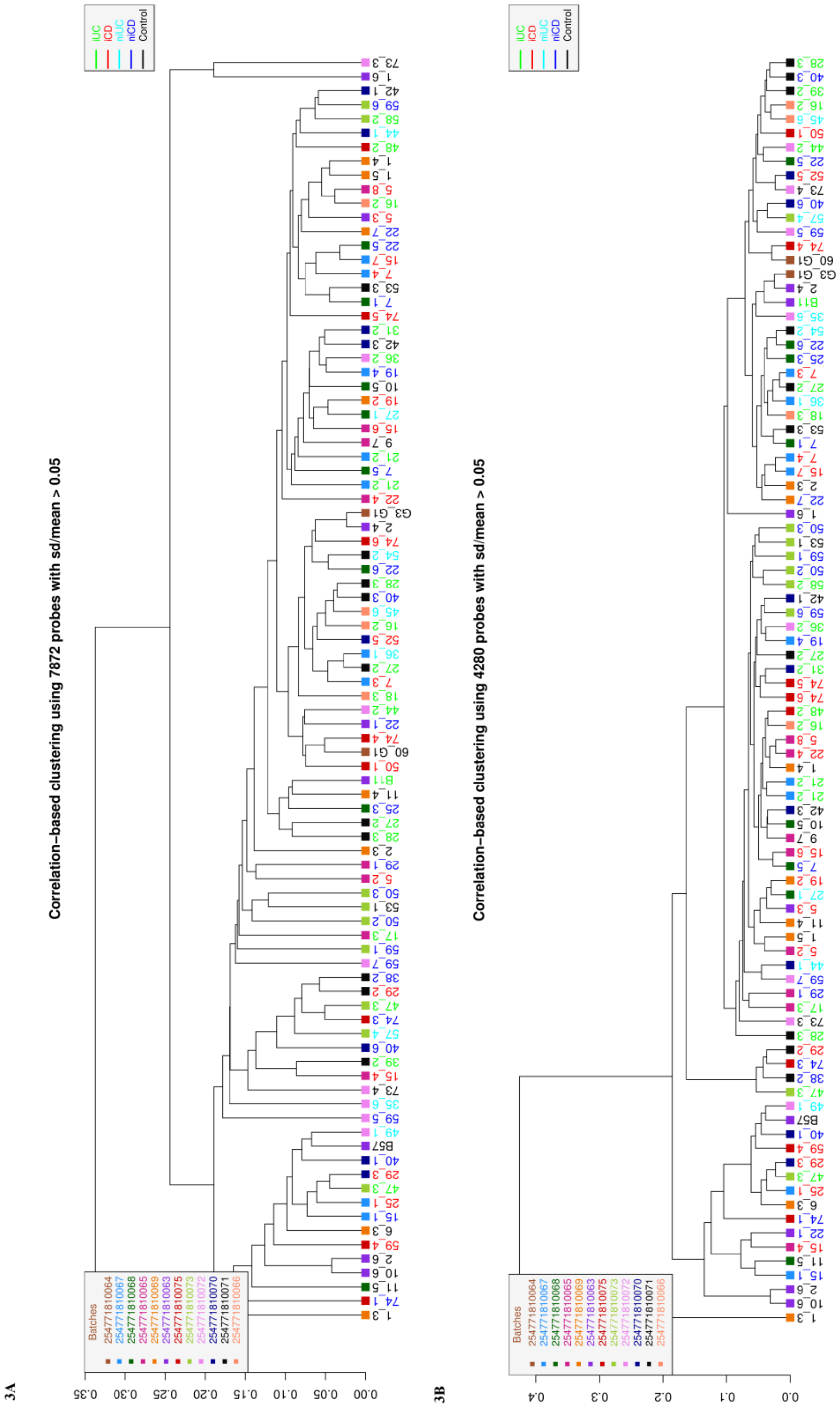

Figure S4

The log2 ratio and –log10 adj.P-values plotted and represented as volcano plots for the non-inflamed tissues contrasts iCD vs niCD (S4A) and iUC vs niUC (S4B). The probes in red, blue and orange colors represents up-regulated (FC >1.5 and adj.P-value <0.05), down-regulated (FC <-1.5 and adj.P-value <0.05) and significant with small fold change (FC >-1.5 and <1.5), respectively. The non-significant probes are represented in black color.

4A

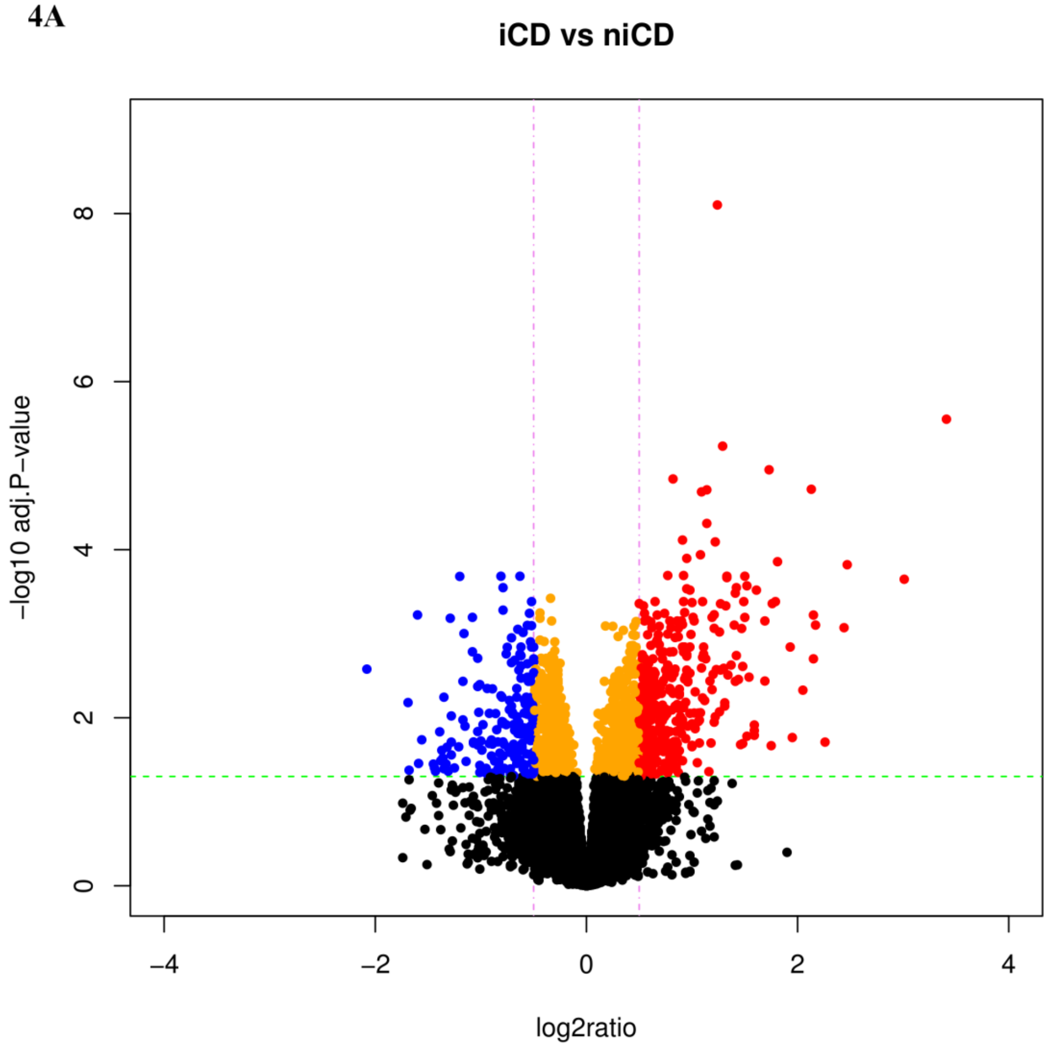

4B

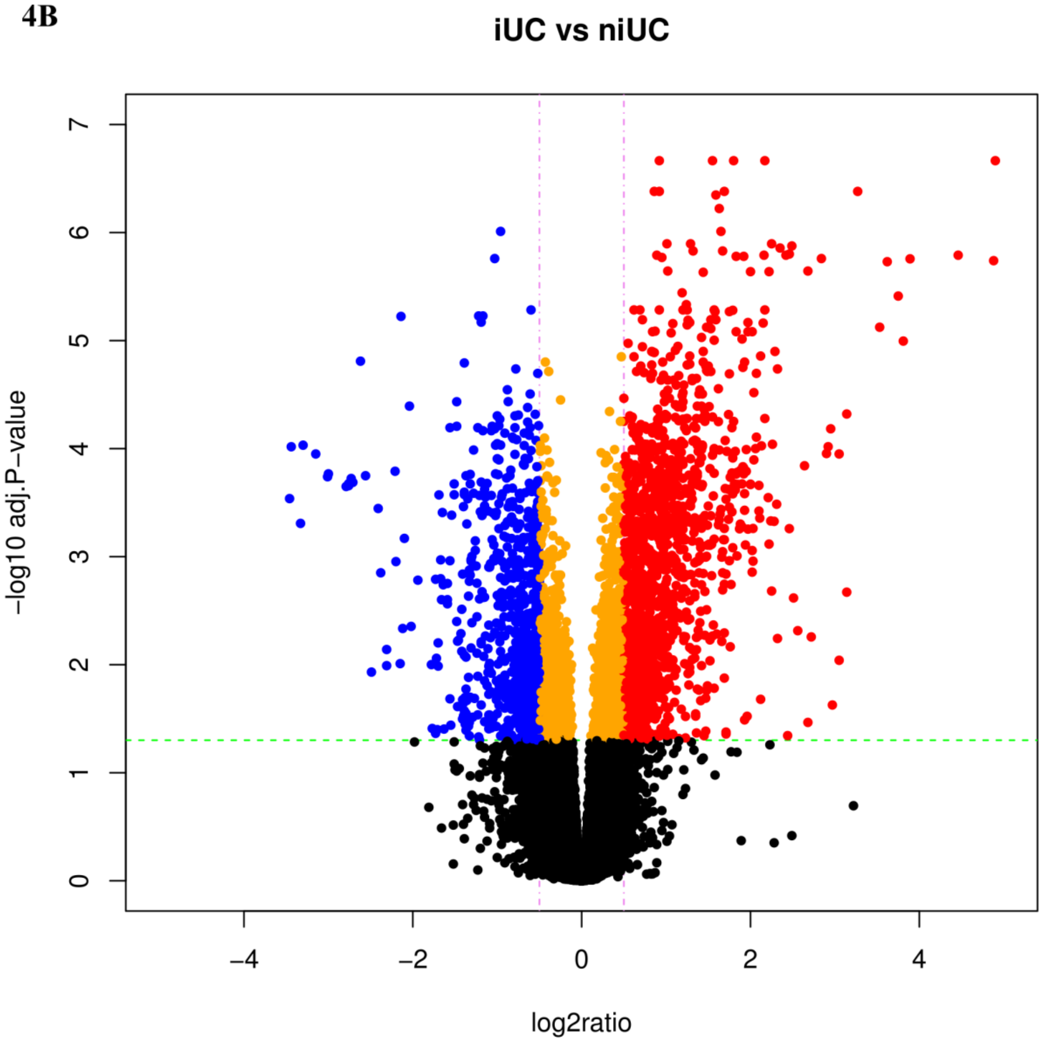

Figure S5

The expression map of the top 40 differentially expressed lncRNAs and protein-coding genes in iCD vs iUC (patients in red, controls in blue) based on unsupervised hierarchical clustering. The clustering algorithm was unable to distinguish between the iCD and iUC samples. The log2 normalized expression values are shown in the color key.

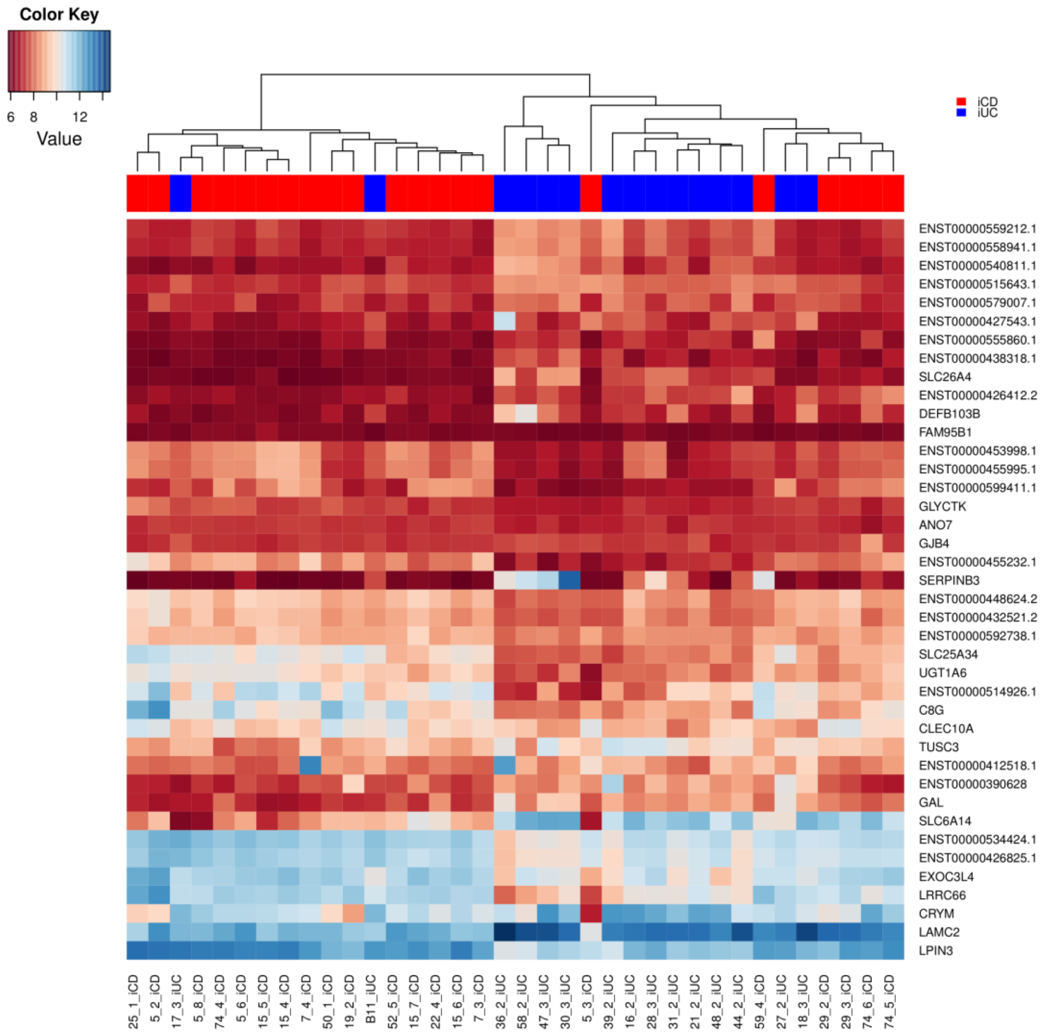

Figure S6

The expression map of the total differentially expressed lncRNAs and protein-coding genes in iCD vs controls (S6A) and iUC vs controls (S6B) (patients in red, controls in blue) based on unsupervised hierarchical clustering. The log2 normalized expression values are shown in the color key.

6A

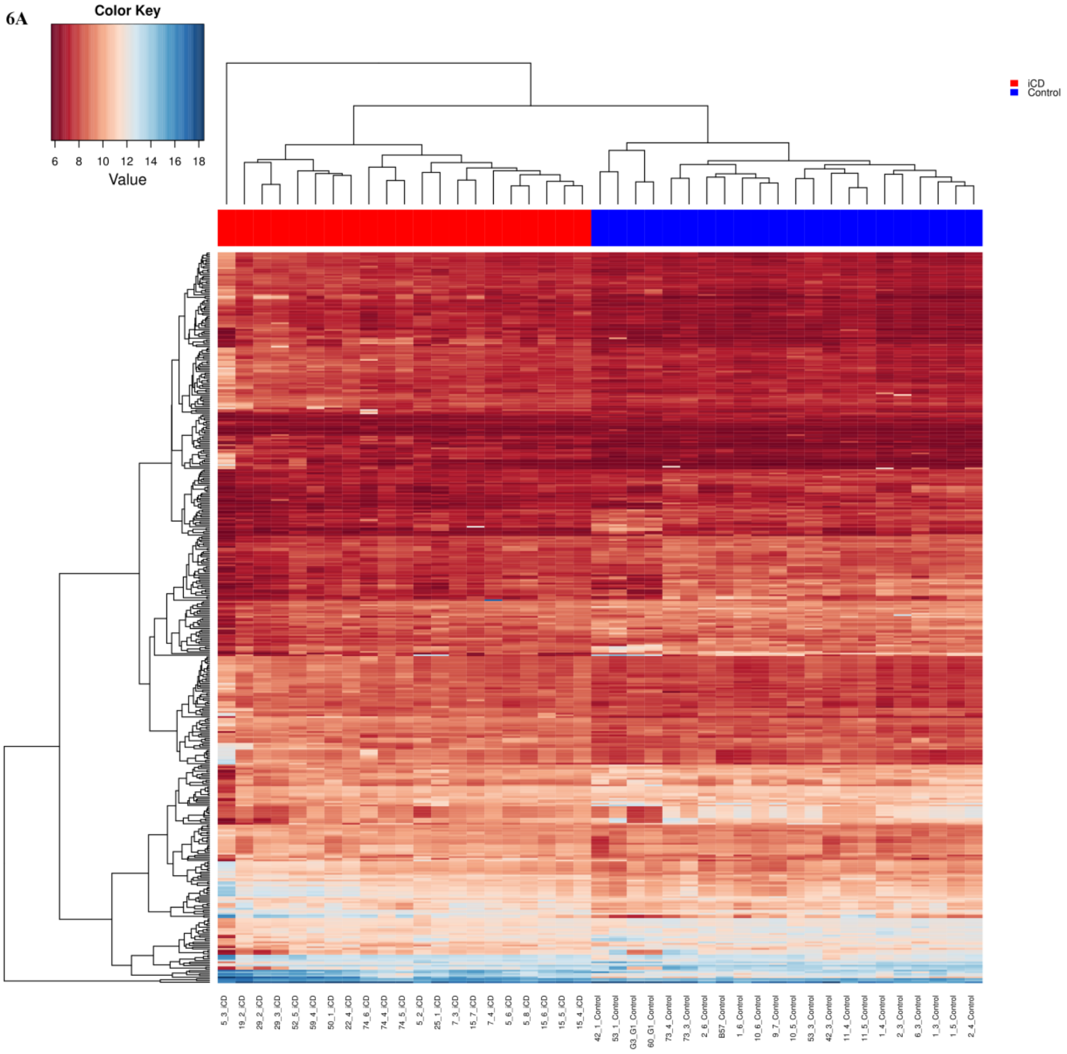

6B

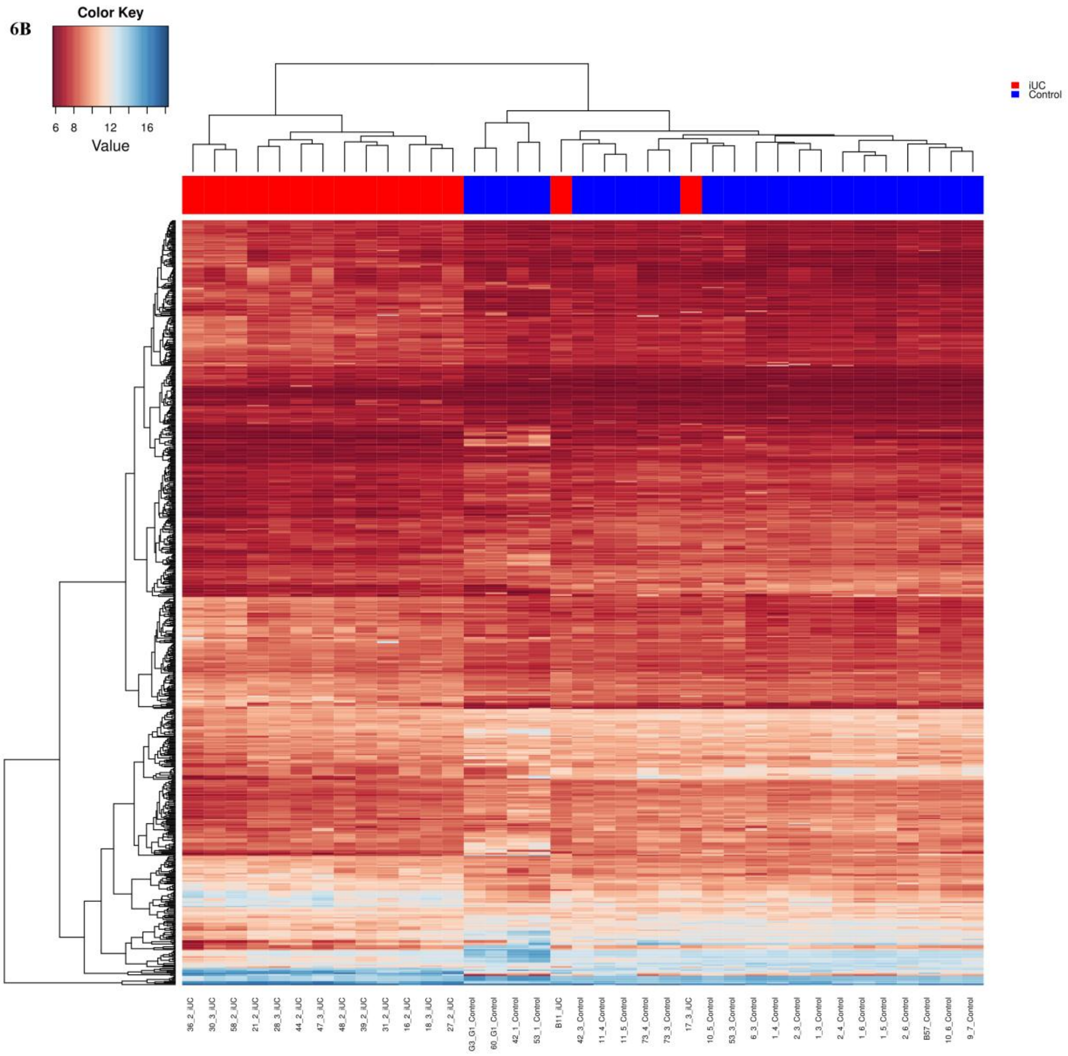
Color Key
6
8
12
16
Value
iUC
Control
36_2_iUC
30_3_iUC
58_2_iUC
21_2_iUC
28_3_iUC
44_2_iUC
47_3_iUC
48_2_iUC
39_2_iUC
31_2_iUC
16_2_iUC
18_3_iUC
27_2_iUC
G3_G1_Control
60_G1_Control
42_1_Control
53_1_Control
B11_iUC
42_3_Control
11_4_Control
11_5_Control
73_4_Control
73_3_Control
17_3_iUC
10_5_Control
53_3_Control
6_3_Control
1_4_Control
2_3_Control
1_3_Control
2_4_Control
1_6_Control
1_5_Control
2_6_Control
B57_Control
10_6_Control
9_7_Control

Figure S7

Dendrogram of samples and heatmap of clinical parameters. Linear regression model and weighted correlation network analysis (WGCNA) were used to investigate the impact of clinical parameters of samples on disease diagnosis.

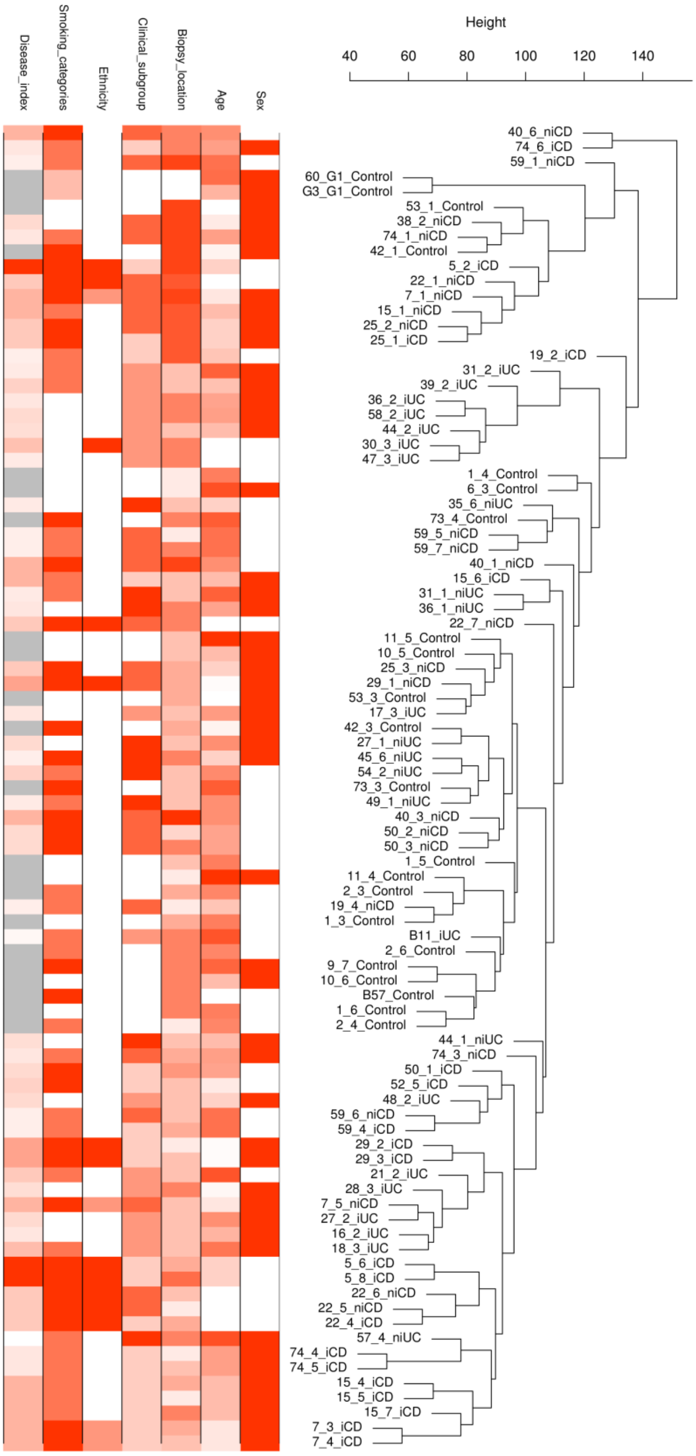

Figure S8

Receiver operating characteristic (ROC) curve analysis for age (a), sex (b), disease index (c), smoking (d) classification using differentially expressed lncRNAs in all five contrasts. ROC analysis for randomly selected same number of lncRNAs (e) was also performed to rule out any selection bias in our analysis.

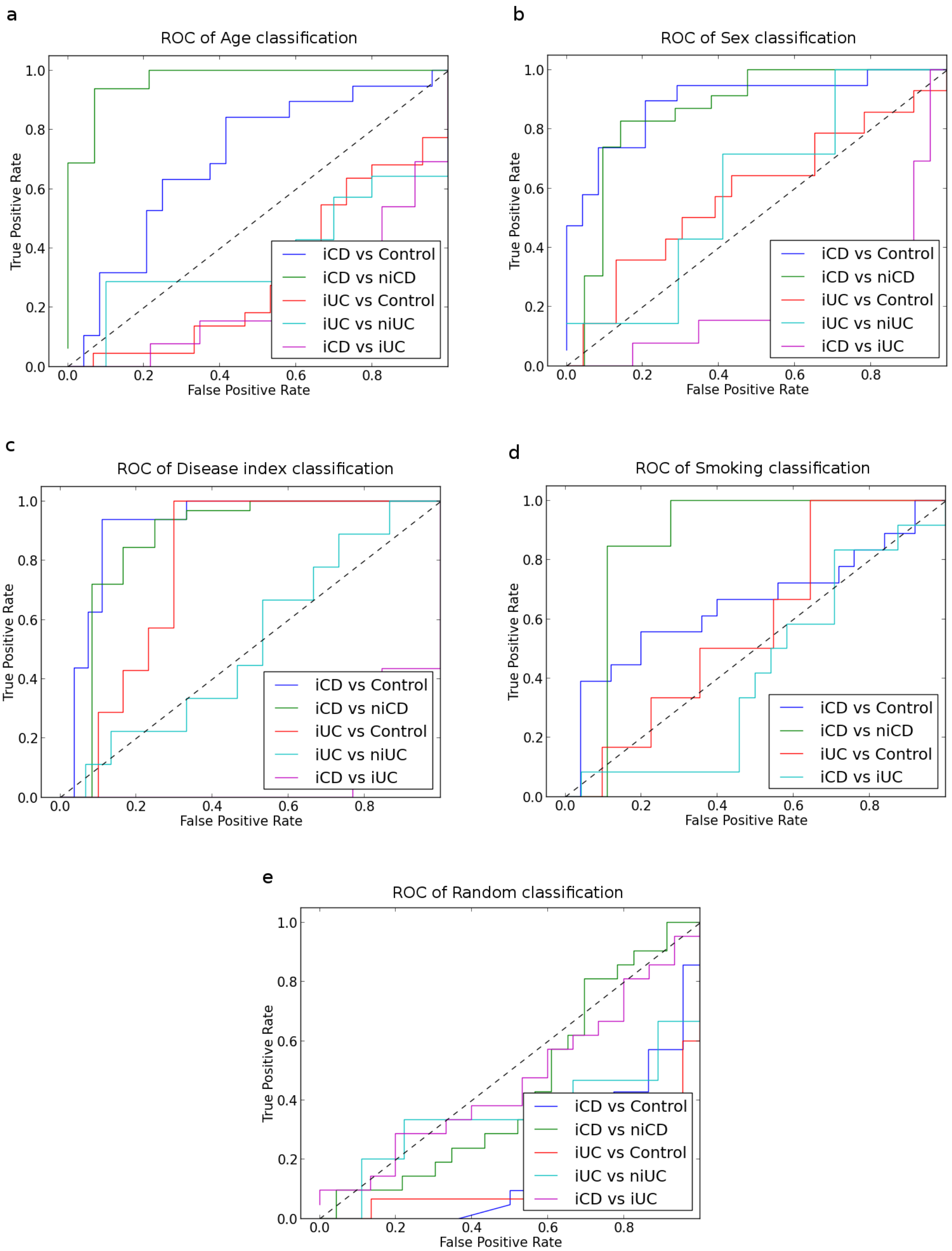

Figure S9

Overlap of differentially expressed genes identified by LIMMA and SVM. SVM was used to cross-validate the differentially expressed genes identified by LIMMA.

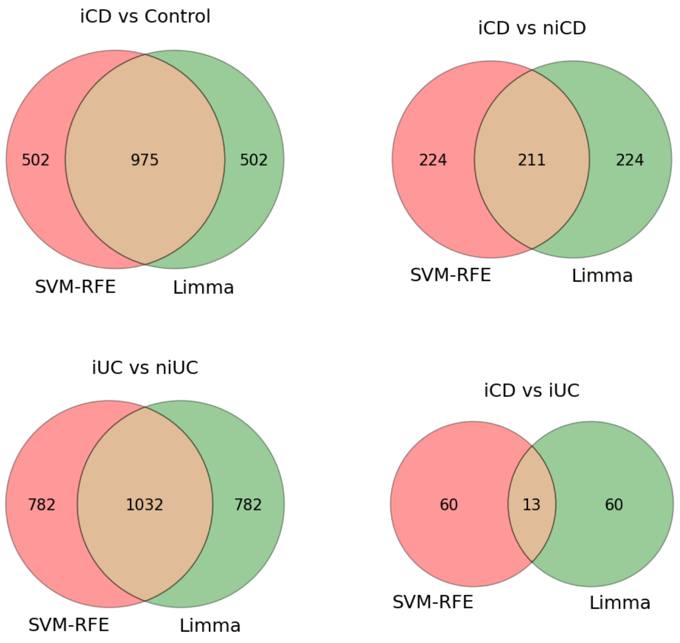

Figure S10

Co-expression network is built by hierarchical clustering and Dynamic Tree Cut. Modules are clusters of highly interconnected genes. The “brown”, “green” and “red” modules are enriched for differentially expressed genes between iUC / iCD and Control.

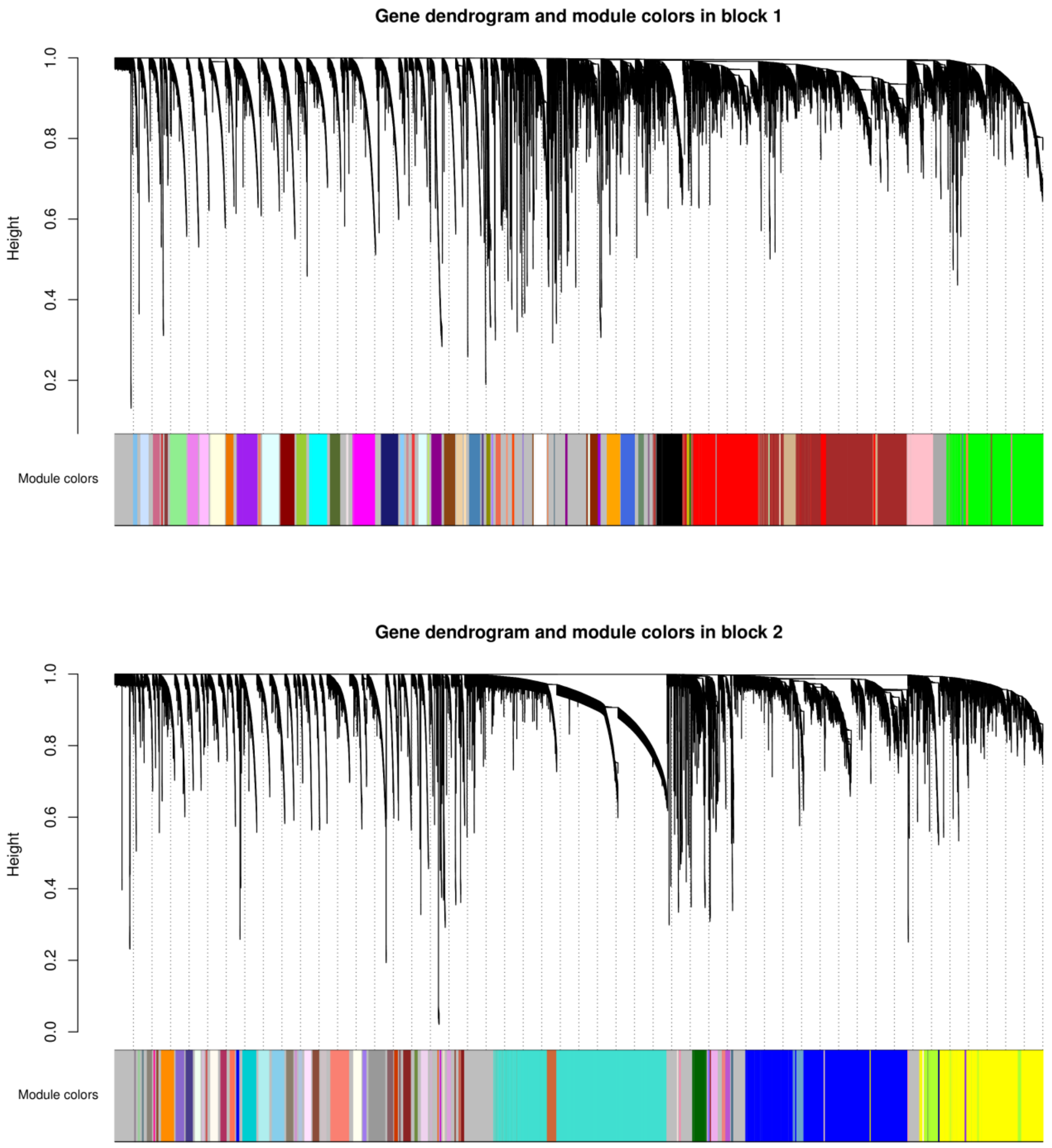

Figure S11

Module significance is determined as the average absolute gene significance measure for all genes in a given module

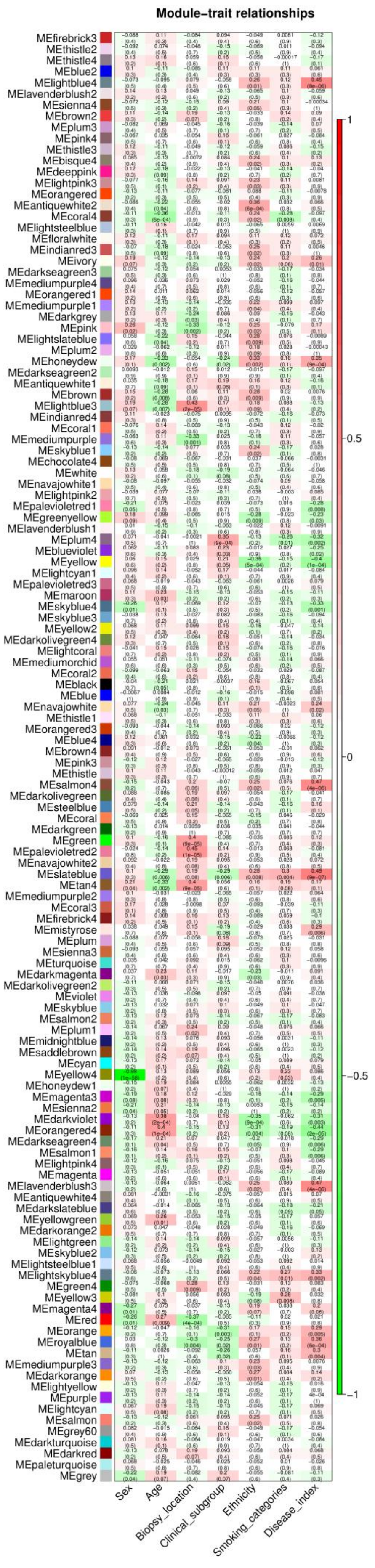

Supplement: Additional file 2: Figure S1. — Pearson correlations calculated for the technical replicates (six samples analyzed in duplicates on separate chips: 16_2, 18_3, 27_2, 28_3, 47_3 and 21_2). Figure S2. Scatterplot matrices describing the variation explained by the first four principal components for 90 biopsy samples. Figure S3. Unsupervised hierarchical clustering of the most dynamic probes (coefficient of variance >0.05) targeting lncRNAs (S3A) and protein-coding genes (S3B) across the samples in different clinical subgroups. Figure S4. Log2 ratio and -log10 adjusted P-values plotted and represented as volcano plots for the non-inflamed tissue comparisons iCD versus niCD (S4A) and iUC versus niUC (S4B). Figure S5. Expression map of the top 40 differentially expressed lncRNAs and protein-coding genes in iCD versus iUC based on unsupervised hierarchical clustering. Figure S6. Expression map of the total differentially expressed lncRNAs and protein-coding genes in iCD versus controls (S6A) and iUC versus controls (S6B) (patients in red, controls in blue) based on unsupervised hierarchical clustering. Figures S7, S8 and S9. Figure S7. Dendrogram of samples and heatmap of clinical parameters. Linear regression model and weighted correlation network analysis (WGCNA) were used to investigate the impact of clinical parameters on disease diagnosis. Figure S8. Receiver operating characteristic (ROC) curve analysis for age (a), sex (b), disease index (c), smoking (d) classification using differentially expressed lncRNAs in all five comparisons. Figure S9. Overlap of differentially expressed genes identified by LIMMA and SVM. Figures S10 and S11. Figure S10. Co-expression network is built by hierarchical clustering and Dynamic Tree Cut. Modules are clusters of highly interconnected genes. The ‘brown’, ‘green’ and ‘red’ modules are enriched for differentially expressed genes between iUC/iCD and control. Figure S11. Module significance is determined as the average absolute gene significance measure for all genes [file 13073_2015_162_MOESM2_ESM.pdf]
